# Δ-9-Tetrahydrocannabinol treatment during adolescence and alterations in the inhibitory networks of the adult prefrontal cortex in mice subjected to perinatal NMDA receptor antagonist injection and to postweaning social isolation

**DOI:** 10.1038/s41398-020-0853-3

**Published:** 2020-06-01

**Authors:** Clara Garcia-Mompo, Yasmina Curto, Hector Carceller, Javier Gilabert-Juan, Esther Rodriguez-Flores, Ramon Guirado, Juan Nacher

**Affiliations:** 1 Neurobiology Unit, Department of Cell Biology, Interdisciplinary Research Structure for Biotechnology and Biomedicine (BIOTECMED), Universitat de Valencia, Valencia, Spain; 2 CIBERSAM: Spanish National Network for Research in Mental Health, Valencia, Spain; 3 Fundación Investigación Hospital Clínico de Valencia, INCLIVA, Valencia, Spain

**Keywords:** Molecular neuroscience, Schizophrenia

## Abstract

The prefrontal cortex (PFC) continues its development during adolescence and alterations in its structure and function, particularly of inhibitory networks, have been detected in schizophrenic patients. Since cannabis use during adolescence is a risk factor for this disease, our main objective was to investigate whether THC administration during this period might exacerbate alterations in prefrontocortical inhibitory networks in mice subjected to a perinatal injection of MK801 and postweaning social isolation. This double-hit model (DHM) combines a neurodevelopmental manipulation and the exposure to an aversive experience during early life; previous work has shown that DHM mice have important alterations in the structure and connectivity of PFC interneurons. In the present study we found that DHM had reductions in prepulse inhibition of the startle reflex (PPI), GAD67 expression and cingulate 1 cortex volume. Interestingly, THC by itself induced increases in PPI and decreases in the dendritic complexity of somatostatin expressing interneurons. Both THC and DHM reduced the density of parvalbumin expressing cells surrounded by perineuronal nets and, when combined, they disrupted the ratio between the density of puncta expressing excitatory and inhibitory markers. Our results support previous work showing alterations in parameters involving interneurons in similar animal models and schizophrenic patients. THC treatment does not modify further these parameters, but changes some others related also to interneurons and their plasticity, in some cases in the opposite direction to those induced by the DHM, suggesting a protective effect.

## Introduction

Schizophrenia is a complex neuropsychiatric disorder characterized by a range of cognitive, behavioral, and emotional dysfunctions^1^. These symptoms usually start between late adolescence and early adulthood^2^, by the time the prefrontal cortex (PFC) ends its development. This neocortical area is related to sensorimotor gating, which is altered in patients with schizophrenia and can be measured by the prepulse inhibition of startle reflex (PPI) test^3^. Structural alterations, such as volumetric changes^4,5^ and decreases in dendritic spine density in the pyramidal neurons^6^ have also been described in the PFC of patients with schizophrenia. Interestingly, in addition to these alterations in excitatory neurons, different neuropathological studies have revealed an important role of inhibitory prefrontocortical circuits in the pathogenesis of schizophrenia^7^. Studies on both patients and animal models have found alterations, particularly in the subpopulation of interneurons expressing parvalbumin (PV)^8–10^. Many of these cortical PV expressing interneurons are surrounded by perineuronal nets (PNNs): specialized regions of the extracellular matrix, which play an important role in the plasticity and maturation of these interneurons^11^. PNNs are also altered in schizophrenic subjects and thus, they may represent an important factor in the pathophysiology of this disorder^12^.

These alterations in excitatory and inhibitory circuits may be the result of genetic predisposition^13^, but environmental factors operating during early life, especially cannabis consumption during adolescence, may be particularly relevant to the emergence of schizophrenia^14^. There are evidences indicating that cannabis use during adolescence can produce impairments in PFC-dependent cognitive tasks during adulthood^15^. Other neurological processes involving the PFC, such as the PPI response, are also reduced after the chronic use of cannabis in humans^16^. Additionally, rodent studies with Δ-9-tetrahydrocannabinol (THC), the main psychoactive compound of cannabis, have reported a reduction in PPI responses^17^. Interestingly, cannabis exposure also modulates prefrontocortical GABAergic function^18^ through the cannabinoid receptor 1 (CB1R)^19^. CB1R are expressed in the presynaptic terminals of basket cells expressing cholecystokinin (CCK)^20^ and are important for the maturation of the inhibitory circuits of the PFC^21^. Postmortem studies have also reported alterations of CB1R expression in the frontal cortex of patients with schizophrenia^22,23^.

Taken together these studies on the effects of cannabis, and particularly the possibility that its use during adolescence may constitute a predisposing factor for schizophrenia, we hypothesize that the administration of THC during rodent puberty may lead to persistent behavioral changes and long-term adaptations of interneuronal structure, connectivity and plasticity in the mPFC. Thus, here we evaluate, using behavioral, molecular and histological approaches, the effects of THC administration during adolescence on mice subjected to a previously described double-hit model (DHM), which combines a single injection of MK801 (NMDAR antagonist) at P7 and postweaning social isolation^24,25^. MK801 treatment is aimed to alter transiently some processes occurring during the last stages of the neurodevelopment, which particularly affect the maturation of the PFC^26^ and results in the long term in subtle modifications of prefrontocortical circuits, particularly on PV+ interneurons^27,28^. On the other hand, social isolation is a chronic stressor intended to simulate adverse experiences during early life in humans. Both paradigms show behavioral, functional and structural alterations similar to some found in schizophrenic patients^29–33^. We have previously shown that the combination of these paradigms in the DHM induces alterations in anxiety and locomotor behaviors and in different parameters related to inhibitory circuits in the PFC^24,25^.

## Material and methods

### Animals, housing, and pharmacological treatments

Sixty-four GIN male mice (Tag [GadGFP] 45704Swn; Jackson Laboratory; Bar Harbor, Maine, USA)^34^ were used in this study. Details on the election of sample size, randomization, blinding and compliance with ethical regulations can be found in the supplementary methods section.

Seven days after birth (P7), male pups received randomly a single intraperitoneal injection of the non-competitive antagonist of the *N*-methyl-d-aspartate (NMDA) receptor, dizocilpine (i.p. MK801, 1 mg/kg; Abcam Biochemicals, Cambridge, UK) or the vehicle solution (NaCl 0.9%) (*n* = 32 per group).

After the injection, pups were returned to their cages and remained with their mother until the age of weaning (P21). At this age, mice from the MK801 injected group were housed alone (social isolation) in small polycarbonate cages (24 × 14 × 13 cm; Zoonlab-Bioscape), constituting the “double-hit” model^24^ (“DHM” group from now on). Animals that received the vehicle were housed in groups of three to four mice (social housing) in standard-size cages (38 × 16 × 13 cm; Zoonlab-Bioscape), constituting the control (CTRL) group. Isolated mice were able to hear and smell other mice but physical or visual contact with them was not allowed.

Then, during the period considered as adolescence in mice, from P28 to P48^35^, half of the animals (chosen randomly) received a daily intraperitoneal injection of Δ-9-tetrahydrocannabinol (THC) (THC Pharm GmbH, Germany) the psychoactive compound of cannabis, at the previously described dose of 10 mg/kg^36^. The other half were injected with vehicle (VEH, 1:1:18 mixture of ethanol:Tween 80®:saline), thus forming the four groups of this experiment (*n* = 16 per group): CTRL-VEH, CTRL-THC, DHM-VEH and DHM-THC, *n* = 16 (Fig. S1).

### Prepulse inhibition of startle reflex test

Starting on P131, all animals were evaluated using the Prepulse Inhibition of Startle Reflex test (PPI) (Fig. S1). Startle responses were measured using the Startle and Fear combined system (Panlab, Barcelona, Spain). A detailed description of the PPI methodology can be found in the supplementary methods section.

#### Fresh tissue extraction and dissection of medial prefrontal cortex

Mice used for gene and protein expression analysis (*n* = 32, 8 animals per group) were sacrificed with sodium pentobarbital (10 ml/kg i.p.) on P133. Brains were immediately removed and placed on Petri dishes filled with cold sterile phosphate buffer (PB). Each hemisphere was stored on separated microcentrifuge tubes, frozen in liquid nitrogen and kept at −80 ^o^C until used. The mPFC from the left and right cortices were dissected in sterile conditions at cold temperature and under RNAse-free conditions.

#### Quantitative retrotranscription-polymerase chain reaction

Total mRNA from mPFC was extracted using RNeasy→ Mini Kit from QIAgen (QIAgen, Germany). Reverse transcription (RT) reactions were performed using Superscript ™ II Reverse Trancriptase (Invitrogen™, Thermo Fischer Scientific, USA). A detailed description of the methodology used can be found in the “Supplementary Methods” section and Table S1.

#### Quantitative immunoblotting

The expression of GAD67 and SYN was evaluated in the dissected mPFC using quantitative immunoblotting with specific antibodies. Details on the experimental protocols can be found in the supplementary methods section.

### Histological procedures

One day after the PPI test (P133), all mice used for histological techniques (*n* = 8 per group) were transcardially perfused under deep pentobarbital anesthesia (1 ml/kg), first for 1 min with NaCl (0.9%) and then with 4% paraformaldehyde.

Brain hemispheres destined to study dendritic arborization and spine density were cut with a vibratome (Leica VT 1000E, Leica; Germany) in 100 µm thick coronal sections. The contralateral brain hemispheres were cryoprotected with 30% sucrose in cold PB 0.1 M (4 °C) for 48 h and then cut in 50 µm coronal sections using a freezing-sliding microtome (Leica SM2010 R, Leica; Germany). Slices were collected in six subseries.

#### Immunohistochemistry

All the studied sections passed through all procedures simultaneously to minimize any difference from immunohistochemical staining itself. All slides were coded and the codes were not broken until the experiment was finished.

Sections were processed “free floating” and were treated for 1 h with 10% normal donkey serum (NDS) (Biowest LLC, Kansas City, USA) in PBS with 0.2% Triton-X100 (Sigma-Aldrich, St. Louis, MO). After this, sections were incubated with different cocktails of two, three or four primary antibodies and *Wisteria floribunda* lectin (see Table S2 for detailed information). After being rinsed, sections were light-protected and incubated 1 h at RT with appropriate secondary antibodies or streptavidin (Table S2). All sections were mounted on slides and coverslipped using DakoCytomation fluorescent mounting medium (Dako North America Inc., Carpinteria, CA).

#### Volumetric analysis

The volumes of the different mPFC regions (prelimbic cortex, PrL; infralimbic cortex, IL and cingulate cortex area 1, Cg1) were measured in sections stained for parvalbumin (PV) and perineuronal nets (PNN), using the “Volumest” plugin in FIJI/ImageJ Software (NIH, USA)^37^, which uses Cavalieri’s principle^38^. Details on image acquisition and analysis can be found in the supplementary methods section.

#### Analysis of dendritic arborization

Dendritic arborization was studied in Cg1, since in PrL and IL the number of EGFP-expressing neurons was very low. Confocal microscopy (Leica TCS SPE, Leica; Germany) was used to obtain z-series of optical sections (0.8 µm apart) covering the dendritic tree of selected interneurons (6 EGFP-expressing neurons per mouse). Details on the requisites for including neurons in the analysis can be found in the supplementary methods section. 3D reconstructions of the neurons were traced using the “Simple neurite tracer” plugin in FIJI software^37^, which also allowed us to analyze their Sholl profile in 3D^39^.

#### Analysis of dendritic spine density

Dendritic spine density was also studied in the cingulate cortex, using confocal microscopy (Leica TCS SPE, Leica; Germany). Individual dendrites were selected from EGFP-expressing neurons in layer III (six neurons per animal). Stacks of confocal images were obtained with a 63×/1.40 oil immersion objective and an additional 3.5 digital zoom. Confocal z-stacks covering the whole depth of the sections were taken with 0.38 μm step size. The stacks were processed with FIJI software^37^, using the “Stitching” plugin to reconstruct a 3D image of apical dendrites. The multipoint tool was used to count the spines in the three dendritic segments (50 μm each) expanding 150 μm from the soma.

#### Analysis of immunoreactive puncta expressing excitatory/inhibitory synaptic markers

We studied the density of puncta expressing vesicular glutamate transporter 1 (VGLUT1) and vesicular GABA transporter (VGAT) in selected confocal planes of different regions of the mPFC (PrL and IL, 1.78 mm Bregma). Confocal z-stacks covering the whole depth of the sections were taken with 1 μm step size and only subsets of confocal planes with the optimal penetration level for each antibody were selected for analysis. On these planes, small regions of the neuropil (505 μm^2^) were selected for analysis, in order to avoid blood vessels and cell somata. Images were processed using customized macros for FIJI software^40–42^. The data were expressed as the number of immunoreactive puncta/μm^2^.

The [number of VGLUT1 + puncta/μm^2^]/[number of VGAT + puncta/μm^2^] has been denominated E/I ratio.

#### Analysis of the density of perisomatic puncta on pyramidal neurons

Sections processed for CaMKII-α, CB1R and SYN immunohistochemistry were observed under a confocal microscope (FV 10i; Olympus, Japan) using a 60x oil objective. The perisomatic puncta on pyramidal neurons were analyzed in the layer III of the different mPFC regions: PrL, IL, and Cg1. The analyses were performed on sections from Bregma 1.94–1.54 mm^43^.

Confocal z-stacks were acquired as described above and images were processed with similar FIJI macros. Details on the selection and analysis of perisomatic puncta can be found in the supplementary methods section. Fifteen neurons per animal and region were analyzed. Finally, values of puncta density for CB1R and SYN were obtained from each neuron and expressed as number of puncta per micron of soma perimeter.

#### Analysis of the density of parvalbumin expressing cells and perineuronal nets

Sections processed for PV immunohistochemistry and the histochemical detection of PNNs were observed under a confocal microscope (Olympus Fluoview FV 10i, Olympus, Japan) using a 10x objective. We estimated the density of cells expressing PV or surrounded by perineuronal nets (PNN) in the different regions of the mPFC: PrL, IL (Bregma +1.78 mm) and Cg1 (Bregma: +0.38 mm)^43^.

### Statistical analyses

Kruskal–Wallis followed by Mann–Whitney post hoc analyses were used to assess the differences in the PPI response because these data did not follow a normal distribution. For the same reason we used Friedman’s test to assess the differences in the PPI response between the three pre-pulse intensities (73, 76, and 82 dB). For all the other analyses, we used two-way ANOVAs with the number of animals as the sample number (n) and with model (CTRL and DHM) and administration (VEH and THC) as between factors. We used three-way ANOVA to evaluate the differences in each of the intersections of the Sholl analysis, using n as the sample number (n) and model, administration and distance from the soma as between factors. The Greenhouse–Geisser test was used when the requirement of sphericity was violated. Post hoc analyses were performed using Bonferroni adjustments. Correlation analyses were performed using Pearson’s correlation coefficient or Spearman’s rho correlation coefficient when the data did not follow a normal distribution. The results are shown as the mean ± the standard error of the mean. All the data were analyzed using the SPSS package, 22.0 version and graphs were created using GraphPad Prism 6.

## Results

### Decrease of prepulse inhibition of startle reflex (PPI) in the “double-hit” model mice

**Fig. 1:**
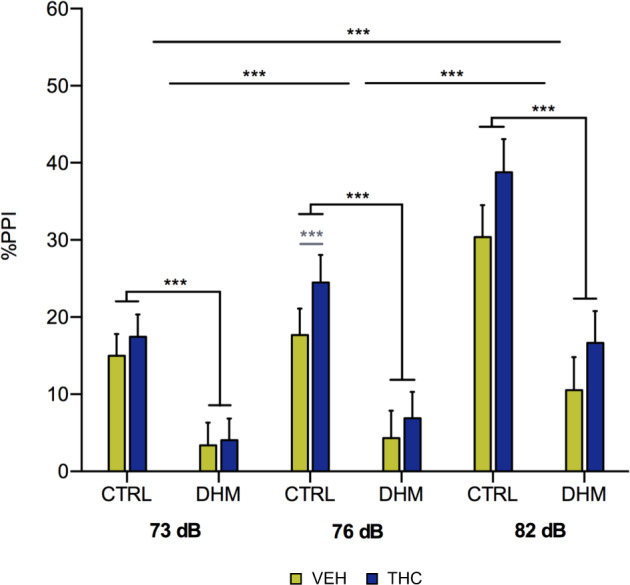
Percentage of PPI in CTRL and DHM animals after VEH or THC administration. Graph representing the percentage of PPI at the three intensities tested, 73, 76, and 82 dB. Black asterisks indicate main effects after Kruskal–Wallis or Friedman’s non-parametric test. Gray symbols and lines in graphs represent statistically significant differences among groups after post hoc analysis. *p* < 0.001 (***). Bars represent the mean ± S.E.M. *n* = 16 animals/group.

First, we analyzed the PPI test to study effects on sensorimotor gating. All mice displayed similar basal startle response of PPI, regardless of treatment factor (VEH, THC) (*p* = 0.524). We found an effect of the animal model on the percentage of PPI response: a decrease at all prepulse intensities (*p* < 0.001) in DHM in comparison with control (CTRL) mice. Friedman’s test revealed an effect of the pre-pulse intensities on the percentage of PPI response (*p* < 0.001). Post hoc analyses showed an increase in the %PPI at 76 dB in CTRL mice injected with THC in comparison with those injected with VEH (*p* = 0.001) (Fig. 1, Table S3).

### Expression of GAD67, CB1R, ErbB4, ST8SiaII, and ST8SiaIV mRNA in the mPFC

To evaluate whether the expression of molecules related to inhibitory neurotransmission and interneuronal plasticity was altered in our experimental conditions, we analyzed the expression of mRNAs from GAD67, ErbB4 and the polysialyltransferases ST8SiaII and ST8SiaIV. We also analyzed the expression of the mRNA of the cannabinoid receptor 1 (CB1R), the main target of THC, which is highly expressed in the presynaptic elements of cholecystokinin+ basket cells. The analysis of the data revealed a significant decrease only in the expression of GAD67 mRNA (*F*(1,28) = 4.436, *p* = 0.044) (Fig. S2A) in DHM mice. No alterations were observed in the rest of genes analyzed (Fig. S2B–E, Table S3).

### Expression of GAD67 and SYN protein in the mPFC

**Fig. 2:**
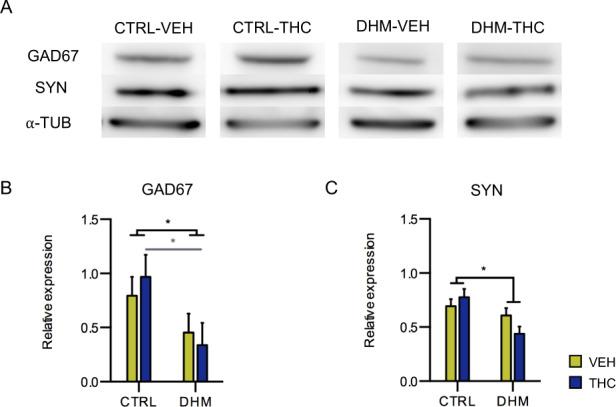
Protein expression studies in the mPFC. Western blot analysis of glutamic acid decarboxylase, 67 kDa isoform (GAD67), synaptophysin (SYN) and α-tubulin (α-TUB) (**a**). Optical density data analyses show a decrease in the expression of GAD67 (**b**) and SYN (**c**) in the DHM model, after two-way ANOVA test. Black asterisks indicate main effects and gray symbols and lines in graphs represent statistically significant differences among groups after post hoc analysis. *p* < 0.05 (*). Bars represent the mean ± S.E.M. *n* = 8 animals/group.

To confirm the alterations found in GAD67 mRNA, we studied the expression of its protein, usually found in presynaptic inhibitory terminals. In addition, we studied the expression of synaptophysin (SYN) to evaluate whether there were changes in the total number of synapses, since its expression is a reliable marker of active synapses^44^. We observed a significant decrease in the expression of GAD67 protein expression in the DHM mice in comparison with CTRL mice (*F*(1,11) = 9.285, *p* = 0.011) (Fig. 2a, b) and SYN (*F*(1,11) = 6.490, *p* = 0.021) (Fig. 2a, c). Post hoc analysis revealed a significant decrease in GAD67 expression in DHM-THC mice in comparison with CTRL-THC mice (*p* = 0.041) (Fig. 2c, Table S3).

### Volume of the mPFC

We hypothesized that these alterations in synaptic markers might influence the total volume of the region. Thus, we analyzed the volume of the different mPFC regions (Figures S3A–C), and found main effects in the volume of the cingulate cortex area 1 (Cg1) in the animal model factor: DHM mice showed a decreased volume when compared to CTRL mice (*F*(1,28) = 5.298, *p* = 0.040) (Fig. S3C). Post hoc analyses did not reveal differences between experimental conditions (Fig. S3, Table S3).

### Structural parameters in GAD-EGFP-expressing interneurons

The decrease in the expression of molecules related to inhibitory neurotransmission in the PFC and its volumetric reduction in DHM animals, might represent alterations in the structure and connectivity of different interneuronal populations. In order to explore whether these morphological changes could be observed in somatostatin expressing interneurons, we used GIN mice, which express EGFP specifically in this subpopulation of inhibitory neurons^34^. Previous studies from our laboratory have determined that in the mPFC most of these EGFP+ cells are somatostatin expressing dendrite-targeting Martinotti cells^45^.

**Fig. 3:**
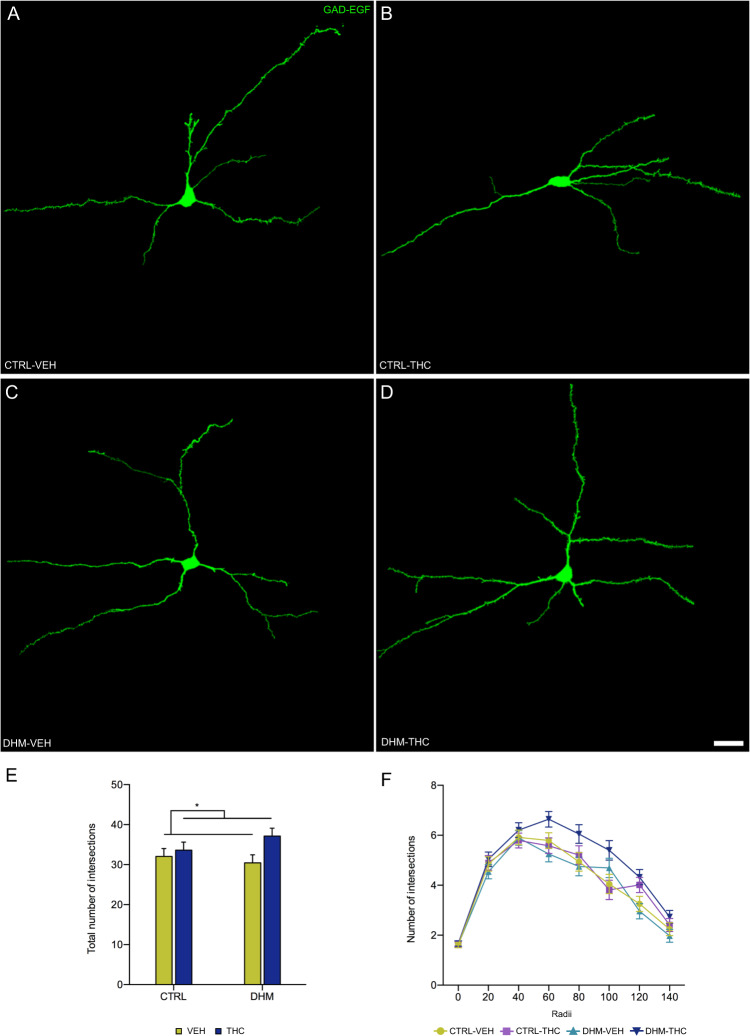
Dendritic arborization in interneurons of the prefrontal cortex. **a–d** Representative images of GAD-EGFP-expressing interneurons showing the dendritic arbor in the four experimental conditions. **e** Graph showing the number of total intersections. **f** Graph showing the number of intersections each 20 micron. Black asterisk in E represents statistically significant effects of THC administration after two-way ANOVA analysis. *p* < 0.05 (*). Bars represent the mean ± S.E.M. Scale bar: 25 μm. *n* = 6 animals/group.

The analysis of their dendritic arborization did not reveal changes due to the animal model, but showed an effect of THC administration (Fig. 3): The total number of dendritic intersections was higher in the THC-treated animals ((*F*(1,28) = 4.358, *p* = 0.046); see Fig. 3e). A three-way ANOVA revealed (i) an effect of THC administration on the number of dendritic intersections with the equidistant Sholl spheres, (*F*(1, 243) = 25.723, *p* < 0.001), being significantly higher in the THC-treated mice; (ii) an effect of the distance from the soma (*F*(8, 243) = 137.101, *p* < 0.001), and (iii) an interaction between the model and administration factors (*F*(1,243) = 9.337, *p* = 0.002). Post hoc analyses only revealed significant differences in the number of intersections between different Sholl spheres, independently of the model and the treatment.

Then, we analyzed the dendritic spine density of this same subpopulation of prefrontocortical interneurons. We found that neither the animal model nor the THC administration affected this parameter (Fig. S4; Table S3).

### Analysis of the density of puncta expressing excitatory and inhibitory synaptic markers

**Fig. 4:**
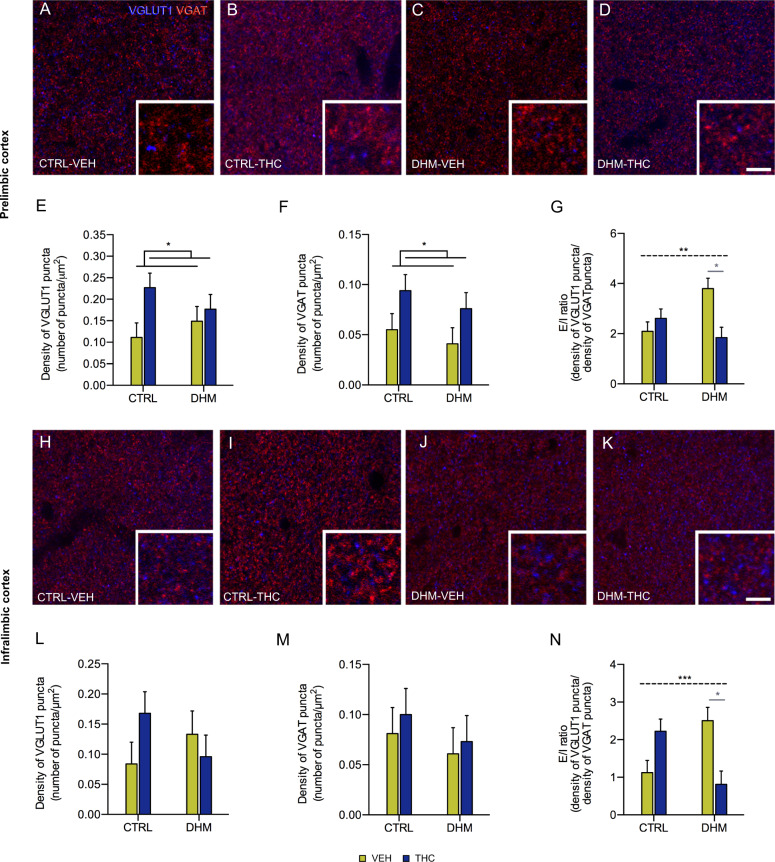
Analysis of markers of excitatory and inhibitory neurotransmission in prelimbic and infralimbic cortices. **a–d** Representative confocal images of excitatory (red, VGLUT1) and inhibitory (blue, VGAT) puncta in the neuropil of prelimbic cortex of CTRL and DHM animals after administration (VEH or THC). Scale bar: 10 and 3.3 μm for the detail. Graphs representing the density of VGLUT1 (**e**) and VGAT (**f**) immunoreactive puncta, and the E/I ratio [number of VGLUT1+ puncta/μm^2^]/[number of VGAT+ puncta/μm^2^] (**g**). **e**, **f** graphs show differences in VGLUT and VGAT expression due to THC administration after two-way ANOVA analysis. Horizontal lines in G represent interaction (black, dashed) after two-way ANOVA. Gray symbols and lines in graphs represent statistically significant differences among groups after post hoc. **h–k** Representative confocal images of excitatory (red, VGLUT1) and inhibitory (blue, VGAT) puncta in the neuropil of Infralimbic cortex of CTRL and DHM animals after administration (VEH or THC). Scale bar: 10 μm and 3.3 μm for the detail. Graphs representing the density of VGLUT1 (**l**) and VGAT (**m**) immunoreactive puncta, and the E/I ratio (**n**). Graphs show differences in the E/I ratio due to the interaction between model and administration after two-way ANOVA analysis *p* < 0.05 (*), *p* < 0.01 (**), *p* < 0.001 (***). Bars represent the mean ± S.E.M. *n* = 6 animals/group.

To better understand the effects of our experimental treatments on prefrontocortical circuitry and the alterations that we have observed in the structure and neurochemistry of interneurons, we analyzed the density of puncta expressing vesicular glutamate transporter 1 (VGLUT1) and vesicular GABA transporter (VGAT) in the mPFC (Fig. 4). The ANOVA revealed a significant effect of THC administration: THC treatment increased the density of VGLUT1 (*F*(1,20) = 4.435, *p* = 0.048) and VGAT (*F*(1,20) = 5.189, *p* = 0.034) expressing puncta in the prelimbic cortex (PrL, Fig. 4a–f). No differences in these parameters were found in the infralimbic cortex (IL, Fig. 4h–m).

We also found a significant interaction between model and administration factors in the E/I ratio (number of VGLUT1+ puncta/μm^2)^/(number of VGAT+ puncta/μm^2^) of PrL (*F*(1,18) = 9.816, *p* = 0.006; Fig. 4g) and IL (*F*(1,18) = 17.179, *p* = 0.001; Fig. 4n). In fact, post hoc analyses showed a decrease in the E/I ratio in DHM-THC mice when compared to DHM-VEH mice in PrL (*p* = 0.024; Fig. 4g) and IL (*p* = 0.013; Fig. 4n).

### Analysis of perisomatic CB1R immunoreactive puncta on pyramidal neurons

To further explore the involvement of cannabinoids and interneurons in our experimental conditions, we analyzed the density of CB1R immunoreactive puncta in the perisomatic region of pyramidal neurons. Here, we studied the density of CB1R positive puncta coexpressing synaptophysin (SYN) to investigate whether those structures corresponded to synaptically active axonal boutons (Fig. S5). THC administration increased significantly the density of CB1R+ puncta on pyramidal neurons of Cg1, (*F*(1,19) = 4.476, *p* = 0.048). We also found a significant interaction between model and administration factors in the density of CB1R positive puncta of the same region (*F*(1,19) = 7.969, *p* = 0.011). In addition, post hoc analysis in this region revealed an increase in the density of CB1R positive puncta in DHM-THC mice when compared to DHM-VEH mice (*p* = 0.018) and CTRL-THC mice (*p* = 0.026); (see Fig. S5G). However, no significant differences were found in the density of SYN+ or CB1R+ /SYN+ puncta (Fig. S5E, Table S3). We did not find significant differences in the density of puncta expressing CB1R or SYN or in their co-localization, neither in the PrL nor in the IL (Fig. S5F, G).

### Density of parvalbumin expressing interneurons and perineuronal nets

**Fig. 5:**
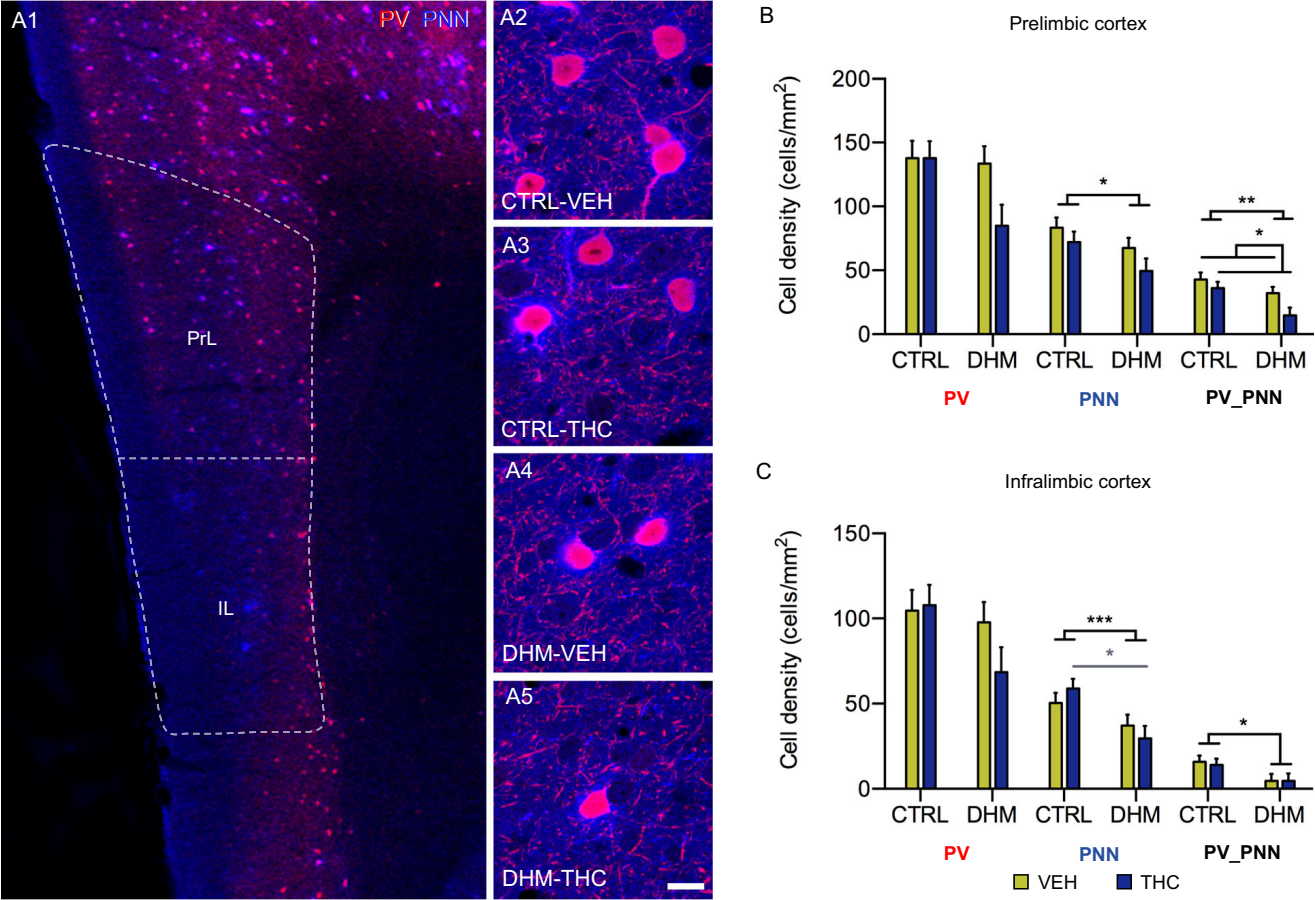
Analysis of the density of PV-immunoreactive neurons and PNNs in PrL and IL cortices. A1, representative confocal images showing the distribution of PV positive cells (red) and PNNs (blue) in the prelimbic (PrL) and infralimbic (IL) cortices. A2–A5 show in detail the co-localization between PV-expressing cells and PNNs. The histogram in B shows a decrease in the density of PNNs in DHM mice and a decrease in the density of PV+ cells surrounded by PNNs due to model and administration of THC in the PrL cortex after two-way ANOVA analysis. The histogram in C shows a decrease in the density of PNNs in DHM mice and a decrease in the density of PV_PNNs in DHM mice in the IL cortex after two-way ANOVA analysis. Black asterisks and lines in graphs represent main effects after two-way ANOVA analysis and grey symbols and lines represent statistically significant differences among groups after post hoc analyses, *p* < 0.05 (*), *p* < 0.01 (**), *p* < 0.001 (***). The columns represent the mean ± S.E.M of cell density (cells/mm^2^). Scale bar: 87 μm for A1; detailed view (A2–A5), 13.5 μm. *n* = 6 animals/group.

Finally, we studied the density of PV expressing interneurons and PNNs, to analyze the effects of our experimental paradigm on these interneurons and the specialized extracellular matrix surrounding them (Fig. 5a). We found that DHM mice had a significant decrease in the density of PNNs (*F*(1,18) = 5.102, *p* = 0.037) and PV expressing interneurons surrounded by PNNs (*F*(1,17) = 9.232, *p* = 0.007) in the PrL (Fig. 5b). Interestingly, THC administration also produced a significant decrease in the density of PV positive interneurons surrounded by PNNs (*F*(1,17) = 5.079, *p* = 0.038) in this region (Fig. 5b and Table S3).

In this same line, in the IL, DHM mice showed a significant decrease in the density of PNNs (*F*(1,17) = 11.327, *p* = 0.004) and of PV expressing interneurons surrounded by PNNs (*F*(1,17) = 6.874, *p* = 0.021) (Fig. 5c). In addition, post hoc analysis in this region revealed a decrease in the density of PNNs in DHM-THC mice in comparison with CTRL-THC mice (Fig. 5c and Table S3). These effects were restricted to the PrL and IL, we did not find any significant effect in the Cg1 (Fig. S6, Table S3).

### Correlation analyses

We have performed correlation analyses using all the parameters of the present study. We found moderate to strong correlations between PPI intensity and GAD67 protein expression in the PFC. Interesting moderate to very strong positive correlations were also found between the expression of the mRNAs of polysialyltransferases, CB1R, ErbB4, and between ST8SiaIV mRNA and the expression of GAD67 protein. There were also positive and moderate correlations between GAD67 protein expression and the density of PNN and PNN surrounding PV+ cells in different regions of the mPFC. Supplemental Tables S4 and S5 show in detail all the results of the correlation analyses performed in our study.

## Discussion

In the present study we describe the effects of THC administration during adolescence in a DHM, combining an early social isolation stress and a perinatal NMDA receptor antagonist treatment, on different behavioral, molecular and structural parameters affecting inhibitory networks in the mPFC. Although none of these specific parameters is further affected in the DHM animals subjected to THC treatment during adolescence, it is interesting to note that this experimental group accumulates most of the behavioral, structural and neurochemical alterations induced by both the model and the THC treatment. It is also very interesting to note that THC by itself also induces changes in parameters related to the inhibitory neurons and in PPI and that in some cases these changes go in the opposite direction to those described in the DHM. This may suggest the presence a “protective effect”, which would be consistent with the hypothesis that subjects with schizophrenia are more likely to consume substances of abuse, such as cannabis, because they may enhance performance in certain tasks. In fact, a recent report has found that lifetime cannabis use is associated with better working memory in patients with schizophrenia-related disorders^46^. Related to this, it is interesting to note that in the present study THC was administered i.p., while it is consumed voluntarily as a drug of abuse in humans.

There is available evidence supporting that the abuse of cannabis during adolescence is a risk factor for the development of schizophrenia^47–50^, notwithstanding the majority of subjects that abuse THC in adolescence do not develop SCZ as adults^51^. It is possible that an interaction between this abuse and a combination of genetic alterations may increase the likelihood of developing the disease. It can also be possible an interaction with aversive experiences affecting the early life of the patients, since these ambient factors are also known to be predisposing factors for schizophrenia. In fact, this is why we have chosen them to become part of our model; the genetic alterations leading to the development of a disorganized circuitry would be modeled, at least partially, with the perinatal MK801 injection^27,28^. Although it would be very interesting to have performed this study also in females, we chose to develop it only in males because it is already a very complex protocol involving a very high number of animals and because schizophrenia has a rate ratio for males:females of 4:1^52^. It is necessary to note that, although the animal model used in our study presents some alterations similar to those found in schizophrenic patients, it has obviously great limitations, because humans are never exposed perinatally to an NMDA receptor antagonist and rarely to severe and prolonged social isolations during early life. In any case, the present results may also be relevant to mood disorders, since social isolation is also considered to model alterations observed in these psychiatric diseases. In fact, we have reported previously alterations in anxiety-related behaviors in the DHM^24^.

We have used PPI as a behavioral readout for the DHM because this test of sensorimotor gating has been used widely used to provide face validity to animal models of schizophrenia: Several studies have shown a reduction of the PPI response in patients, which is strongly associated with positive symptoms^53,54^ and similar reductions have been found previously in animal models of this disease^55,56^. In this line, here we show for the first time that our DHM induces a reduction in the PPI response, in addition to previously reported alterations in anxiety and locomotor behaviors^24^.

It has been suggested that THC consumption might trigger an early onset of schizophrenia in patients with previous vulnerability^57^. Some studies have found that chronic cannabis use leads to a deteriorated PPI response^16,58^, although some other did not find differences in this parameter^59^. In addition, it is known that chronic cannabis consumption produces a decrease of this neurological process in schizophrenic subjects^60,61^ and in animal models subjected to chronic THC administration^17,62^. These results are apparently in contrast with our own, but it has to be considered that these studies in human subjects and experimental rodents were performed in adult individuals, which had been exposed to THC for long periods and the PPI was analyzed without discontinuation of THC exposure.

In our paradigm, THC administration ended up 83 days before the PPI test. We do not know whether PPI was altered in these animals at P43, the end of THC treatment, but if so, this response had reverted to normal levels in adult control individuals and it apparently does not aggravate the effects of the DHM on this parameter. The long washout period after the last dose of THC guarantees that the obtained findings are not masked by an acute effect of the cannabinoid and represent real changes in the structure and connectivity of mPFC interneurons and on sensorimotor gating. We have selected this THC dose (considerably higher than those used by humans) because previous studies have shown that it produces important effects on behavior after its chronic administration^63^.

Since our goal was to explore the effects of our paradigm on prefrontocortical inhibitory networks, we first analyzed the expression of GAD67 mRNA and protein, finding that our results were in accordance with previous reports. Several studies have shown reductions in the expression of GAD67 mRNA and protein in the PFC of schizophrenia patients^10,64–67^ and similar results have been found in another DHM combining a maternal immune activation with restraint stress during adolescence^68^. It is interesting to mention that, as in the present study, previous reports have found simultaneous reductions in the expression of GABAergic markers in the PFC and in PPI^68,69^. In this line, we have observed a positive correlation of these two parameters, suggesting that the effects of the model on behavior may be linked to alterations in the inhibitory networks of the PFC. Similarly, we also found reduced expression of the synaptic protein SYN, in DHM animals. This is in agreement with studies in postmortem material from schizophrenia patients^70^ and in other animal models of this disorder^71^. Previous studies on rodents have reported lower levels of GAD67 in the PFC after THC administration during adolescence, which resulted in a psychotic-like phenotype in adulthood^19^. However, unlike these studies, we did not find changes in GAD67 mRNA or protein expression after THC administration during adolescence. We think that this discrepancy may be due to differences in the age of sacrifice of the animals and the period between the sacrifice and the end of THC administration

Volumetric reductions in the PFC have been described throughout the literature in patients suffering from schizophrenia^4,5^ and also in animal models of the disease^72^. In line with this, we have found a reduction in the volume of Cg1 in the DHM mice, which is also consistent with our previous results on this model^24^. On the other hand, although a reduction in cortical thickness has been described in schizophrenic and bipolar patients with a history of cannabis use^5^, we did not find alterations in mPFC volume after THC treatment during adolescence, neither in control mice nor in those subjected to the DHM.

Volumetric alterations can be the consequence of structural changes involving dendritic arbor complexity and spine density. Many studies have shown alterations in the structure of excitatory neurons both in schizophrenic patients^6^ and animal models of this disorder^73,74^. However, the structure of interneurons has never been studied in the brain of patients and scarcely in animal models. For this reason, we have analyzed dendritic arborization and spine density in somatostatin expressing interneurons of the mPFC^45^. However, we did not find significant differences between DHM and CTRL mice, in accordance with a previous study^24^. In that report we described an increase in the spine density of these interneurons in the DHM, but only a trend towards an increase was found in our present study. When we analyzed the effects of THC on the structure of somatostatin expressing interneurons, we found an increase in the dendritic arborization in the DHM-THC mice in comparison with the DHM-VEH treated mice. Our results go in the opposite direction to those previously described in layer III pyramidal neurons of rats, in which THC administration during the adolescence produces an atrophy in the dendritic arbor^75^. These opposite effects resemble those described in the mPFC after chronic stress, which causes dendritic atrophy in pyramidal neurons^76^ and hypertrophy in somatostatin expressing interneurons^77^.

The excitatory/inhibitory balance is an essential factor in the maturation of the neural circuitry during development^78,79^ and an imbalance towards less inhibition has been described in schizophrenic subjects^80^. Alterations in the ratio between the density of excitatory and inhibitory puncta (E/I ratio) are suggestive of such an imbalance. However, electrophysiological recordings are necessary to confirm this alteration. We found significant decreases in the E/I ratio in the PrL and IL of DHM-THC-treated mice in comparison with DHM-VEH mice, which are long-lasting effects derived from THC administration during adolescence and may contribute to a decreased excitation of prefrontocortical circuits.

THC treatment also increases the density of perisomatic CBR1+ puncta, specifically on Cg1 pyramidal neurons. This is apparently in contrast to a previous study that reported a decrease in the number of CCK+ expressing interneurons after THC administration during adolescence, but this study analyzed the whole PFC^19^. We have not observed changes in the density SYN+/CB1R+ puncta, but it is known that the density of CB1R is significantly higher on preterminal axons than on synaptic terminals^81^.

One of the most important players in the regulation of the development and plasticity of interneurons, especially of those expressing PV, are the PNNs^82,83^. A reduction in the density of PNNs has been described in the PFC of schizophrenic subjects and in rodent models of this disorder^84–86^. Our present results show a decrease in the density of PNNs and also in that of PV-PNNs in PrL and IL of DHM mice, in agreement with the previous literature. Interestingly, we have also described for the first time a reduction in the density of PV interneurons surrounded by PNNs due to THC administration during the adolescence. These alterations in the PNNs surrounding PV+ interneurons found in the model and in THC-treated animals should have an important impact on the connectivity and physiology of these inhibitory neurons. Future studies should be directed to explore whether THC administration during adolescence may induce alterations in the synaptic input and output (specially the perisomatic innervation on pyramidal neurons) or the physiology of PV+ interneurons. Another interesting possibility is whether, since a reduced PV-PNN density has been associated with immature stages of cortical development^87^, the reductions found in our model and after THC treatment may have contributed to an extended vulnerability of this structure to environmental events.

Previous studies from Corfas laboratory^26^ have demonstrated that juvenile social isolation results in alterations in the myelination of the PFC, which cannot be recovered after a critical period. These effects will have an important impact on the physiology of excitatory circuits, which may in turn affect the interneurons regulating them. It is also possible that these isolation-induced effects on myelination affect directly some interneuronal populations, especially PV+ cells, since a substantial proportion of neocortical myelin content is contributed by these inhibitory neurons^88^. In our previous study in mice we found that social isolation by itself has an important impact on prefrontocortical PV+ cells^24^.

In summary, although none of the parameters altered in the DHM is further altered by the administration of THC, this cannabinoid produces alterations in other parameters that add up to the ones induced by the model, which may result in a more disrupted mPFC network. However, THC treatment also induces changes that appear to counteract some of the ones produced by the DHM, suggesting the presence of some protective effects.

## Supplementary information

Supplemental methods, tables and figure legends

Figure S1

Figure S2

Figure S3

Figure S4

Figure S5

Figure S6

Table S4

table S5

tables 4 and 5
