# Supplementary material for: Δ-9-Tetrahydrocannabinol treatment during adolescence and alterations in the inhibitory networks of the adult prefrontal cortex in mice subjected to perinatal NMDA receptor antagonist injection and to postweaning social isolation
Source: Transl Psychiatry. 2020 Jun 1;10:177. doi: 10.1038/s41398-020-0853-3 (PMC7266818; doi:10.1038/s41398-020-0853-3)
Supplement: Supplementary file 1 — Supplemental methods, tables and figure legends [file 41398_2020_853_MOESM1_ESM.docx]

**SUPPLEMENTARY MATERIAL.**

SUPPLEMENTARY METHODS

**Sample size election, randomization blinding and compliance with ethical regulations.**

Sample size for each experiment was chosen based on previous experience, including a study with the same strain and model and aimed to detect a p < 0.05 in the different tests applied. No animals were excluded from the current study. All the experiments and data analyses were performed blind to treatment conditions.

All animal experimentation was conducted in accordance with the Directive 2010/63/EU of the European Parliament and of the Council of 22 September 2010 and was approved by the Committee on Bioethics of the Universitat de València.

**Prepulse Inhibition of Startle Reflex Test.**

Test sessions were preceded by a habituation phase consisting of three sessions in which mice were placed in the apparatus for five minutes without background noise. Test sessions consisted of no stimulus trials, pulse trials, prepulse trials and prepulse pulse trials. Each no-stimulus trial consisted of a background noise only, pulse trials consisted of a 40 ms pulse of 120 decibels (dB), prepulse trial consisted of a 20 ms 73, 76 or 82 dB pulse followed 100 ms later by a 40 ms pulse of 120 dB. Test sessions began with a 5 minute acclimatization period using a background noise of 70 dB. The test sessions consisted of a ten pulse basal trials and ten presentations of four different trials previously described. Trials were presented in pseudorandom order with an intertrial interval (ITI) of 22 seconds and an interstimulus interval of 100 ms. The percentage of prepulse inhibition of startle reflex was calculated using the next formula: 100- {[(startle response for prepulse+pulse)/(startle response for pulse alone)]x100}.

***Quantitative retrotranscription-polymerase chain reaction***

For quantitative retrotranscription-polymerase chain reaction (qRT-PCR) analyses, each sample was run in duplicates. qPCR was carried out with the ABI PRISM 7700 Sequence Detector (Applied Biosystems) using EVA Green PCR master mix (Applied Biosystems), specific primers for all genes (table S1) at a concentration of 240 nm, and 4 µl cDNA (50 ng) of each sample. TATA box-binding protein (TBP), a housekeeping gene, was used as a reference gene. The expression of TBP did not differ between the different experimental groups. Following a 95 °C denaturation for 10 min, the reactions were cycled 40 times with a 95 °C denaturation for 15 s, and a 60 °C annealing step for 1 min. After this, a melt curve was performed to assess the specificity of primers. Primers were designed by Primer Blast free software, between exons to avoid genomic DNA amplification, using Ensemble data sequences. All DNA oligonucleotide primers were custom synthesized by Metabion international AG (Martinsried, Germany). Relative quantification was performed using the comparative threshold (CT) method according to the 2^-DDCt^ method [^1^](https://paperpile.com/c/prigpV/7RUYE). Changes in gene expression were reported as fold changes relative to controls.

***Quantitative immunoblotting***

Sixty micrograms of total protein for glutamic acid decarboxylase, 67 kDa isoform (GAD 67) and 30 for synaptophysin (SYN) were separated from each sample on 10% SDS–PAGE and transferred to Hybond enhanced chemiluminescence (ECL) nitrocellulose membranes (GE Healthcare). After saturation of nonspecific sites with blocking buffer (5% BSA in PBS-0.025% Tween 20, 2 hours, RT), membranes were incubated overnight at 4°C with primary antibodies against GAD67 (1:1000, Abcam), SYN (1:5000, Sigma-Aldrich) or the loading control protein α-tubulin (1:2000, Sigma-Aldrich), diluted in blocking buffer. After washing with PBS-0.025% Tween 20, membranes were incubated for 2 h at RT with secondary horseradish peroxidase-linked antibodies (1:1500, Sigma-Aldrich), and finally developed using ECL detection reagents (GE Health care). Bands were detected using ImageQuant LAS 4000 system (GE Health care) and densitometry analysis on every band was calculated using FIJI/ImageJ software[^2^](https://paperpile.com/c/prigpV/6qix7). The expression of α-tubulin did not differ between the different experimental groups. SYN and GAD67 densitometry values were normalized to within-lane α-tubulin. Every sample was immunoblotted in duplicate and mean ± SEM was then calculated.

**Volumetric analysis**

Images of all slices were acquired with a confocal microscope (Olympus FV-10; Olympus, Japan). After that, the measures of the areas were estimated in images containing: PrL (Bregma 3.08 mm to 1.70 mm); IL cortex (Bregma 1.94 mm to 1.42 mm) or Cg1 (Bregma 2.34 mm to − 0.22 mm).

**Analysis of dendritic arborization**

In order to be analyzed, EGFP-expressing neurons had to fulfill the following features: 1- the dendritic arbor of the cell must show at least a process with a length greater than 120 μm and 2- the soma must be located at least 30 μm deep from the surface of the tissue.

**Analysis of the density of perisomatic puncta on pyramidal neurons**

The soma profile of the pyramidal neurons was drawn manually and then the selection was enlarged 1 μm in order to include the puncta closely surrounding the somata. A puncta was defined as having an area not smaller than 0.15 and not larger than 2.5 μm^2^ ^3^

**References**

1. [Pfaffl MW. A new mathematical model for relative quantification in real-time RT-PCR. *Nucleic Acids Res* 2001; **29**: e45.](http://paperpile.com/b/lzOkZl/Ime8)

2. [Schindelin J *et al.* Fiji: an open-source platform for biological-image analysis. *Nat Methods* 2012; **9**: 676–682.](http://paperpile.com/b/lzOkZl/1HMCM)

3. [Di Cristo G *et al.* Activity-dependent PSA expression regulates inhibitory maturation and onset of critical period plasticity. Nature Neuroscience. 2007; **10**: 1569–1577.](http://paperpile.com/b/lzOkZl/EcJc)

**SUPPLEMENTARY FIGURE LEGENDS AND TABLES**

**SUPPLEMENTARY FIGURE LEGENDS**

**Figure S1. Schematic representation of the experimental design.** Abbreviations: P, postnatal; CTRL, control group; DHM, “double-hit” model; VEH, vehicle; THC, Δ-9-tetrahydrocannabinol; PPI, prepulse inhibition of startle reflex; Hab, habituation.

**Figure S2. Gene expression studies in the mPFC.** Graphs representing mRNA fold change (A-E).  Data analyses show statistically differences in the expression of GAD67 (A) due to DHM. No differences in the expression of CB1R (B), ErbB4 (C), STS8sia II (D) and STS8siaIV (E) after two-way ANOVA test. p<0.05 (*). Bars represent the mean ± S.E.M. n=8 animals/group

**Figure S3. Volumetric analysis of the mPFC.** Graphs representing the volume of the prelimbic (A) infralimbic (B) and cingulate, area 1 (Cg1) (C) cortices. Volumetric analysis shows a decrease in the volume of Cg1 in DHM mice. Asterisks represent statistically significant effects after two-way ANOVA analysis. p<0.001 (***). Bars represent the mean ± S.E.M. n=6 animals/group.

**Figure S4. Dendritic spine density in interneurons of the prefrontal cortex.** A-D) Representative image of spiny dendrites from GAD-EGFP expressing interneurons. E & F) Analysis of the dendritic spine density in total 150 μm (E) and in the 3 different 50 μm segments (F). Graph shows no differences due to model and administration factors after two-way ANOVA analysis. Bars represent mean ± S.E.M. Scale bar: 5μm and 1.7 μm for the detail. n=6 animals/group

**Figure S5. Analysis of the perisomatic puncta on pyramidal neurons of the cingulate area 1, infralimbic and prelimbic cortices.** A1-D1) Confocal planes of the cingulate cortex, area 1 of the different experimental groups showing SYN and CB1R immunoreactive puncta surrounding CAMKII expressing somata. A2-D2) CaMKII, A3-D3) CB1r and A4-D4) SYN expression in CTRL and DHM animals after the administration of VEH or THC. Graphs representing the density of perisomatic puncta (puncta/mm) expressing SYN, CB1r and its co-localization in the cingulate cortex, area 1 (E), prelimbic cortex (F) and infralimbic cortex (G). Black asterisk and lines in graphs represent main effects after two-way ANOVA analysis, black asterisk and dashed line represent interaction after two-way ANOVA analysis and grey symbols and lines represent statistically significant differences among groups after *post-hoc* analyses, p<0.05 (*). Bars represent the mean ± S.E.M. Scale bar: 2 μm. n=6 animals/group.

**Figure S6. Analysis of the density of PV-immunoreactive neurons and PNNs in Cg1** cortex. A1, representative confocal images showing the distribution of PV (red) and PNNs (blue) in the Cg1 cortex. A2-A5 show in detail the co-localization between PV-expressing cells and PNNs.  The histogram in B shows the density of PV-expressing cells, the density of PNNs and PV-PNN co-localization in CTRL and DHM animals after administration (VEH or THC) in Cg1 cortex. The columns represent the mean ± S.E.M of cell density (cells/mm^2^). Scale bar: 87 μm for A1; detailed view (A2-A5), 13,5 μm. n=6 animals/group.

**SUPPLEMENTARY TABLES**

**Table S1.** Sequences of gene specific primers and associated amplicon lengths for qRT-PCR. (1) Amplicon length in base pairs.

| Target gene | Primers | Sequence (5′ → 3′) | Amplicon size^(1)^ |
| --- | --- | --- | --- |
| GAD67 | Forward | CTGGAGCTGGCTGAATACCT | 120 |
|  | Reverse | TCGGAGGCTTTGTGGTATGT |  |
| CB1r | Forward | TGTCCCTCACCCTGGGCACC | 134 |
|  | Reverse | TCCCAGGAGATCGGCCACCG |  |
| ErbB4 | Forward | CAGTCGCCCAGGGTGCAACG | 133 |
|  | Reverse | GCGAACACTGTGGGGTCGGC |  |
| ST8SiaII | Forward | GGCAACTCAGGAGTCTTGCT | 123 |
|  | Reverse | GTCAGTCTTGAGGCCCACAT |  |
| ST8SiaIV | Forward | CCTTCATGGTCAAAGGAGGA | 125 |
|  | Reverse | CCAGTAACCTCTGACCGCAT |  |
| TATA BP | Forward | CACTTCGTGCAAGAAATGCTG | 89 |
|  | Reverse | AATCAACGCAGTTGTCCGTG |  |

**Table S2.** Primary and secondary antibodies used in this study.

| Primary antibodies | | | | | |
| --- | --- | --- | --- | --- | --- |
| Anti- | Host | Isotype | Dilution | Incubation | Company |
| CAMKIIα | Mouse | IgG1 | 1:500 | 48 hours, 4ºC | Abcam |
| CB1r | Rabbit | IgG | 1:1000 | 24 hours, RT | Synaptic Systems |
| GFP | Chicken | IgY | 1:1000 | 24 hours, RT | Abcam |
| PV | Guinea pig | IgG | 1:2000 | 24 hours, RT | Synaptic Systems |
| SYN | Guinea pig | IgG | 1:1000 | 24 hours, RT | Synaptic Systems |
| VGAT | Rabbit | IgG | 1:500 | 24 hours, RT | Synaptic Systems |
| VGLUT1 | Guinea pig | IgG | 1:2000 | 24 hours, RT | Abcam |
| WFA Lectin biotin conjugated * | Wisteria Floribunda | - | 1:200 | 24 hours, RT | Sigma |
| Secondary antibodies | | | | | |
| Anti- | Host | Label | Dilution | Incubation | Company |
| Chicken IgY | Goat | CF 488 | 1:400 | 1 hour, RT | Sigma |
| Rabbit IgG | Donkey | Cy3 | 1:400 | 1 hour, RT | Jackson Inmunoresearch |
| Streptavidin |  | Alexa Fluor 647 | 1:400 | 1 hour, RT | Invitrogen |
| Mouse IgG1 | Donkey | Dylight 405 | 1:400 | 1 hour, RT | Jackson Inmunoresearch |
| Rabbit IgG | Donkey | AlexaFluor 555 | 1:400 | 1 hour, RT | Invitrogen |
| Guinea pig IgG | Goat | Alexa Fluor 647 | 1:400 | 1 hour, RT | Invitrogen |
| Guinea pig IgG | Donkey | Cy3 | 1:400 | 1 hour, RT | Jackson Inmunoresearch |
| Rabbit IgG | Donkey | Alexa Fluor 647 | 1:400 | 1 hour, RT | Molecular Probes |

**Abbreviations**: CaMKIIα, α subunit of the Ca^2+^ /calmodulin dependent protein kinase II; CB1r, cannabinoid receptor 1; GFP, green fluorescent protein; PV, parvalbumin; SYN, synaptophysin; VGAT, vesicular γ-aminobutyric acid (GABA) transporter; VGLUT1, vesicular glutamate transporter 1; WFA, Wisteria Floribunda.

RT, room temperature.

* WFA is used to detect chondroitin sulphate proteoglicans in perineuronal nets (PNNs). This type of molecules can be recognized by the N-acetylgalactosamine-(GalNac-) binding plant lectin Wisteria Floribunda agglutinin.

**Table S3.** Summary of results

| **parameter** | **Main effects (two-way ANOVA, three-way ANOVA, Kruskal Wallis or Friedman’s test)** | | | | **Group differences (Bonferroni post-hoc unless otherwise stated)** |
| --- | --- | --- | --- | --- | --- |
|  | **model** | **administration** | **Other factor (specify if required)** | **interaction** |  |
| **behaviour** |  |  |  |  |  |
| *% PPI* |  |  | **Prepulse intensities (third factor in Friedman’s test)** |  |  |
| 73dB | ↓(1) | - | **(4)** | - | - |
| 76dB | ↓  (2) | - | 76dB>73dB | - | B>A *** (Mann-Whitney) |
| 82dB | ↓ (3) | - | 82dB>76dB 82dB>73dB | - | - |
| **Gene expression** |  |  |  |  |  |
| CB1r | - | - |  | - | - |
| GAD67 | ↓ (5) | - |  | - | - |
| ErbB4 | - | - |  | - | - |
| SIA II | - | - |  | - | - |
| SIA IV | - | - |  | - | - |
| **Protein expression** |  |  |  |  |  |
| GAD67 | ↓ (6) | - |  | - | B>D * |
| SYN | ↓ (7) | - |  | - | - |
| **Brain volume** |  |  |  |  |  |
| PrL | - | - |  | - | - |
| IL | - | - |  | - | - |
| Cg1 | ↓ (8) | - |  | - | - |
| **Structure of interneurons** |  |  |  |  |  |
| *Dendritic arborization* |  |  | **Distance from the soma (third factor in a three way ANOVA analysis)** | **Interaction model x administration** |  |
| total | - | ↑ (9) |  | - | - |
| 0 | - | ↑(10) | ↑ (11) | ↑ (12) | - |
| 20 | - |  |  |  | - |
| 40 | - |  |  |  | - |
| 60 | - |  |  |  |  |
| 80 | - |  |  |  | - |
| 100 | - |  |  |  | - |
| 120 | - |  |  | - |  |
| 140 | - |  |  | - | - |
| *Dendritic spines* |  |  |  |  |  |
| total | - | - |  | - | - |
| 50 | - | - |  | - | - |
| 100 | - | - |  | - | - |
| 150 | - | - |  | - | - |
| **Density of neuropil puncta** |  |  |  |  |  |
| **PrL** |  |  |  |  |  |
| VGLUT1 | - | ↑ (13) |  | - | - |
| VGAT | - | ↑ (14) |  | - | - |
| **IL** |  |  |  |  |  |
| VGLUT1 | - | - |  | - | - |
| VGAT | - | - |  | - | - |
| **Excitation/inhibition ratio** |  |  |  |  |  |
| PrL | - | # (15) |  | ↓ (16) | C>D * |
| IL | - | - |  | ↓ (17) | C>D * |
| **Perisomatic inervation** |  |  |  |  |  |
| **PrL** |  |  |  |  |  |
| CB1R | - | - |  | - | - |
| SYN | # (18) | - |  | - | - |
| CB1R-SYN | - | - |  | - | - |
| **IL** |  |  |  |  |  |
| CB1R | - | - |  | - | - |
| SYN | - | - |  | - | - |
| CB1R-SYN | - | - |  | - | - |
| **Cg1** |  |  |  |  |  |
| CB1R | - | ↑ (19) |  | ↑ (20) | D>C*  D>B* |
| SYN | - | - |  | - | - |
| CB1R-SYN | - | - |  | - | - |
| **PV+ neurons** |  |  |  |  |  |
| PrL | # (21) | - |  | - | - |
| IL | ↓ (22) |  |  |  | - |
| Cg1 | - | - |  | - |  |
| **PNNs** |  |  |  |  |  |
| PrL | ↓ (23) | - |  | - |  |
| IL | ↓ (24) |  |  | - | B>D * |
| Cg1 | - | - |  | # (25) |  |
| **PV-PNNs colocalization** |  |  |  |  |  |
| PrL | ↓ (26) | ↓ (27) |  | - | B>D # |
| IL | ↓ (28) |  |  | - |  |
| Cg1 | - | - |  | - | - |

Symbols: (↓) statistically significant decrease (Kruskal-Wallis), (↑) statistically significant increase or (↓) decrease (two-way or three-way ANOVA); (-) no statistically significant effect (two-way or three-way ANOVA); **p* < 0.05; ***p* < 0.01; ****p* < 0.001; # 0.10 ≥ *p* ≥ 0.05; A (CTRL-VEH), B (CTRL-THC), C (DHM-VEH), D (DHM-THC).

F and p-values: (1) p<0.001; (2) p<0.001; (3) p<0.001; (4) p<0.001; (5) F(1,28)=4.436, p=0.044; (6) F(1,11)=9.285, p=0.011; (7) F(1,11)=6.490, p=0.021; (8) F(1,28)=5.298, p=0.040; (9) F(1,28)=4.358, p=0.046; (10) (F(1, 243)= 25.723, p<0.001); (11) (F(8, 243)= 137.101, p<0.001); (12) F(1,243)=9.337, p=0.002); (13) F(1,20)=4.435, p=0.048; (14) F(1,20)=5.189, p=0.034; (15) F(1,18)=3.318, p=0.085; (16) F(1,18)=9.816, p=0.006; (17) F(1,18)=17.179, p=0.001; (18) F(1,20)=3.474, p=0.077; (19) F(1,19)=4.476, p=0.048; (20) F(1,19)=7.969, p=0.011; (21) F(1,18)=4.070, p=0.059; (22) F(1,18)=3.288, p=0.087; (23) F(1,18)=5.102, p=0.037; (24) F(1,17)=11.327,p=0.004; (25) F(1,19) =3.466, p=0.079; (26) F(1,17)=9.232, p=0.007; (27) F(1,17)=5.079, p=0.038; (28) F(1,17)=6.874, p=0.018.

**Table S4**. Spearman’s Rho correlation analysis between prepulse inhibition of startle reflex at different intensities (73, 76 and 82 dB) and other variables.

- Bold values indicate: **correlation is significant at the 0.01 level (2-tailed); * correlation is significant at the 0.05 level (2-tailed).

**Table S5.** Pearson’s correlation analysis of the different molecular and histological variables studied.

- Bold values indicate: **correlation is significant at the 0.01 level (2-tailed); * correlation is significant at the 0.05 level (2-tailed).
