# Supplementary material for: Δ-9-Tetrahydrocannabinol treatment during adolescence and alterations in the inhibitory networks of the adult prefrontal cortex in mice subjected to perinatal NMDA receptor antagonist injection and to postweaning social isolation
Source: Transl Psychiatry. 2020 Jun 1;10:177. doi: 10.1038/s41398-020-0853-3 (PMC7266818; doi:10.1038/s41398-020-0853-3)
Supplement: Supplementary file 2 — Figure S1 [file 41398_2020_853_MOESM2_ESM.pptx]

## Slide 1
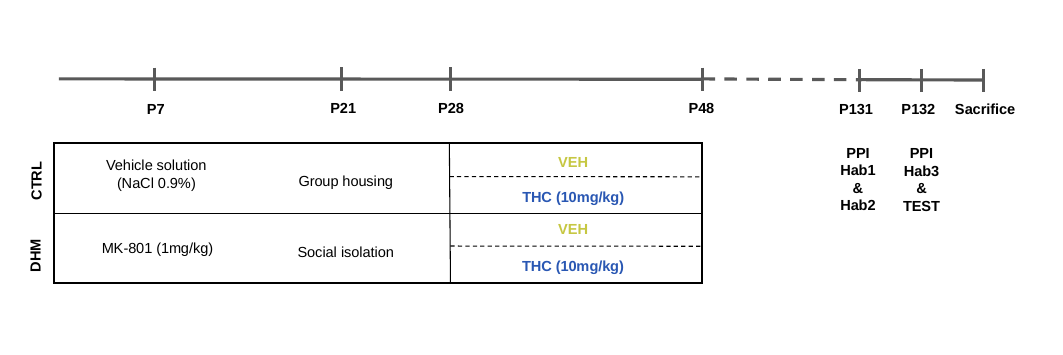

P48
P21
P28
P7
P131
P132
Sacrifice
PPI
Hab1
&
Hab2
PPI
Hab3
&
TEST
VEH
Vehicle solution (NaCl 0.9%)
CTRL
Group housing
THC (10mg/kg)
VEH
MK-801 (1mg/kg)
Social isolation
DHM
THC (10mg/kg)
