# Supplementary figures and images for: Δ-9-Tetrahydrocannabinol treatment during adolescence and alterations in the inhibitory networks of the adult prefrontal cortex in mice subjected to perinatal NMDA receptor antagonist injection and to postweaning social isolation
Source: Transl Psychiatry. 2020 Jun 1;10:177. doi: 10.1038/s41398-020-0853-3 (PMC7266818; doi:10.1038/s41398-020-0853-3)

## Slide 1
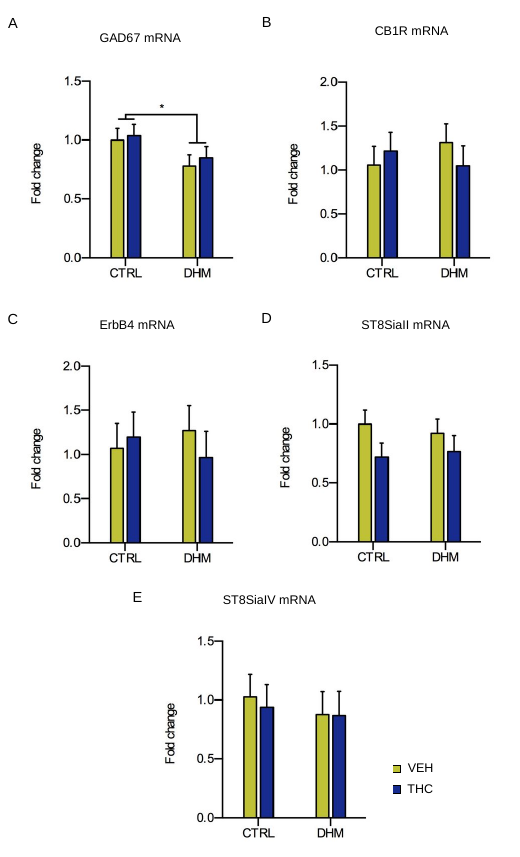

B
A
CB1R mRNA
GAD67 mRNA
D
C
ST8SiaII mRNA
ErbB4 mRNA
E
ST8SiaIV mRNA
VEH
THC

Supplement: Supplementary file 3 — Figure S2 [file 41398_2020_853_MOESM3_ESM.pptx]

## Slide 1
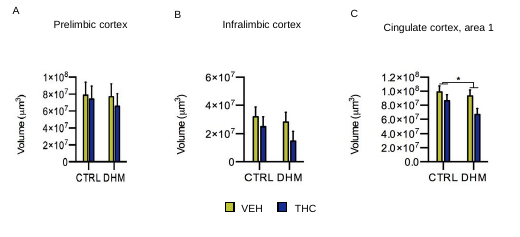

C
A
B
Infralimbic cortex
Prelimbic cortex
Cingulate cortex, area 1
VEH
THC

Supplement: Supplementary file 4 — Figure S3 [file 41398_2020_853_MOESM4_ESM.pptx]
