# Supplementary material for: Δ-9-Tetrahydrocannabinol treatment during adolescence and alterations in the inhibitory networks of the adult prefrontal cortex in mice subjected to perinatal NMDA receptor antagonist injection and to postweaning social isolation
Source: Transl Psychiatry. 2020 Jun 1;10:177. doi: 10.1038/s41398-020-0853-3 (PMC7266818; doi:10.1038/s41398-020-0853-3)
Supplement: Supplementary file 5 — Figure S4 [file 41398_2020_853_MOESM5_ESM.pptx]

## Slide 1
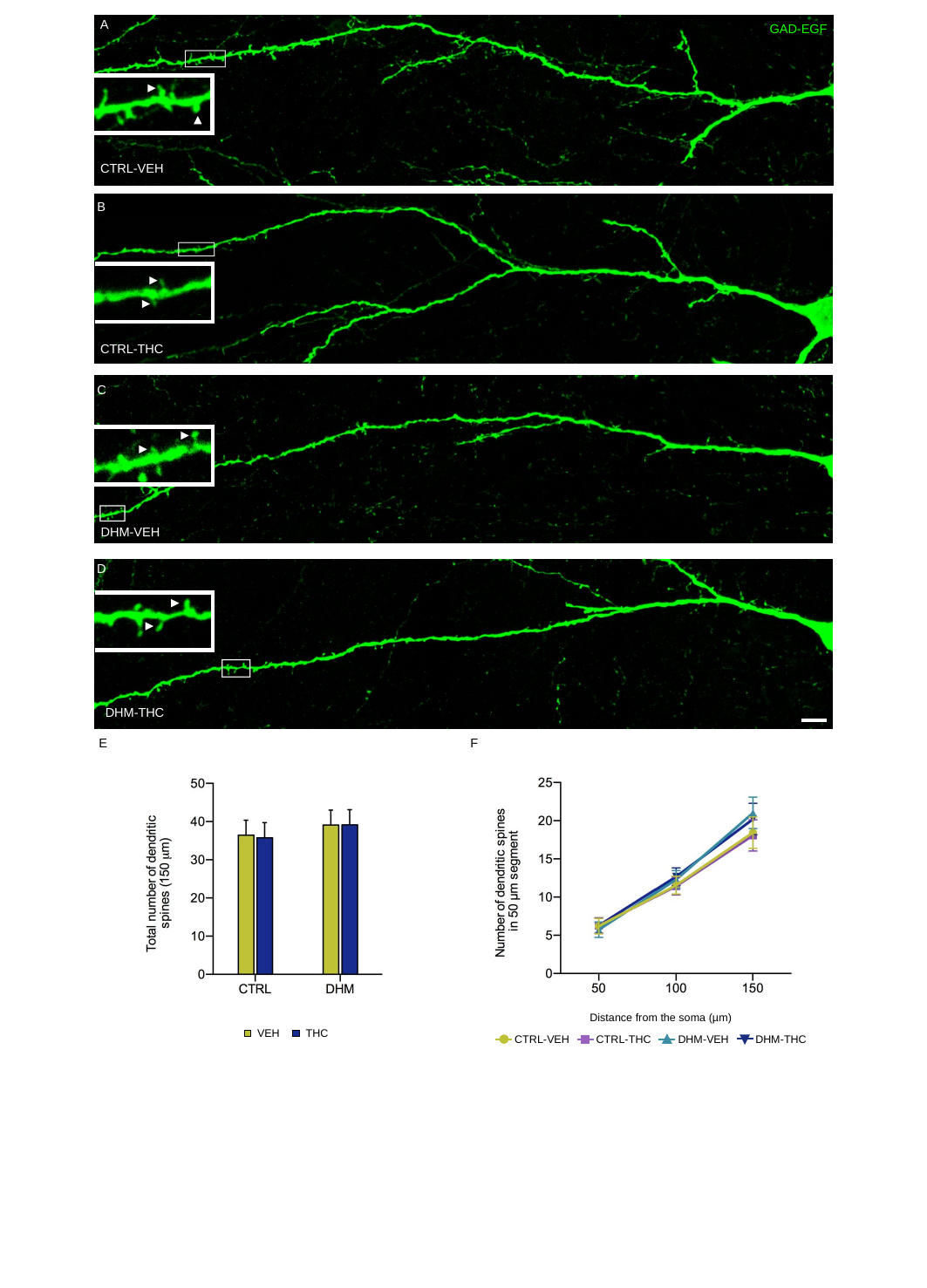

A
GAD-EGF
CTRL-VEH
B
CTRL-THC
C
DHM-VEH
D
DHM-THC
F
E
Distance from the soma (µm)
VEH
THC
CTRL-THC
CTRL-VEH
DHM-VEH
DHM-THC
