# Supplementary material for: Δ-9-Tetrahydrocannabinol treatment during adolescence and alterations in the inhibitory networks of the adult prefrontal cortex in mice subjected to perinatal NMDA receptor antagonist injection and to postweaning social isolation
Source: Transl Psychiatry. 2020 Jun 1;10:177. doi: 10.1038/s41398-020-0853-3 (PMC7266818; doi:10.1038/s41398-020-0853-3)
Supplement: Supplementary file 6 — Figure S5 [file 41398_2020_853_MOESM6_ESM.pptx]

## Slide 1
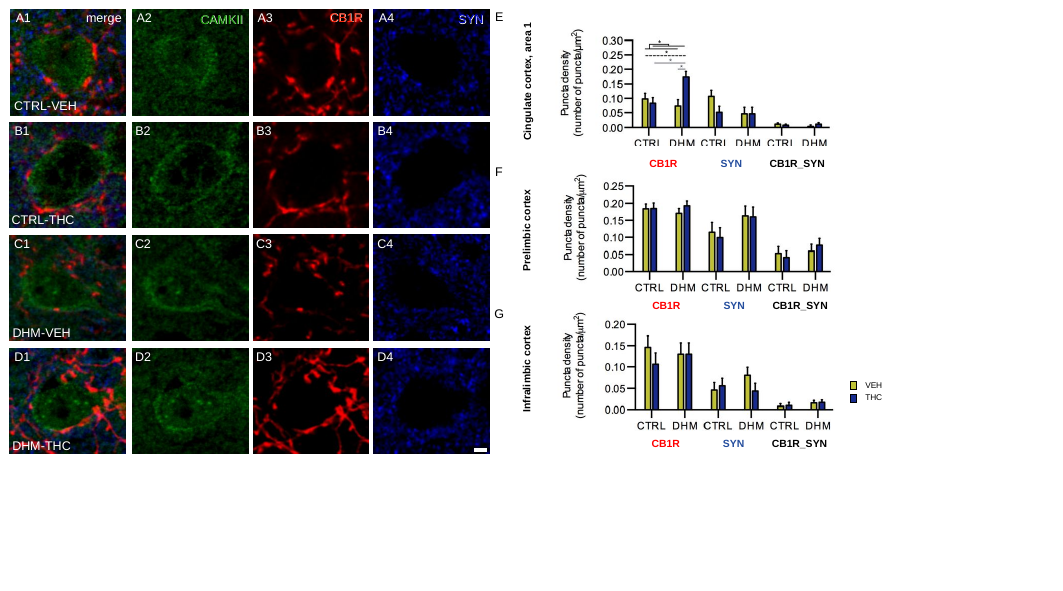

E
A1
merge
A2
A3
CB1R
CB1R
A4
SYN
CAMKII
SYN
CAMKII
Cingulate cortex, area 1
CTRL-VEH
B1
B2
B3
B4
CB1R
CB1R_SYN
SYN
F
CTRL-THC
Prelimbic cortex
C1
C2
C3
C4
CB1R
CB1R_SYN
SYN
G
DHM-VEH
D1
D2
D3
D4
Infralimbic cortex
VEH
THC
CB1R
CB1R_SYN
SYN
DHM-THC
