# Supplementary material for: Δ-9-Tetrahydrocannabinol treatment during adolescence and alterations in the inhibitory networks of the adult prefrontal cortex in mice subjected to perinatal NMDA receptor antagonist injection and to postweaning social isolation
Source: Transl Psychiatry. 2020 Jun 1;10:177. doi: 10.1038/s41398-020-0853-3 (PMC7266818; doi:10.1038/s41398-020-0853-3)
Supplement: Supplementary file 7 — Figure S6 [file 41398_2020_853_MOESM7_ESM.pptx]

## Slide 1
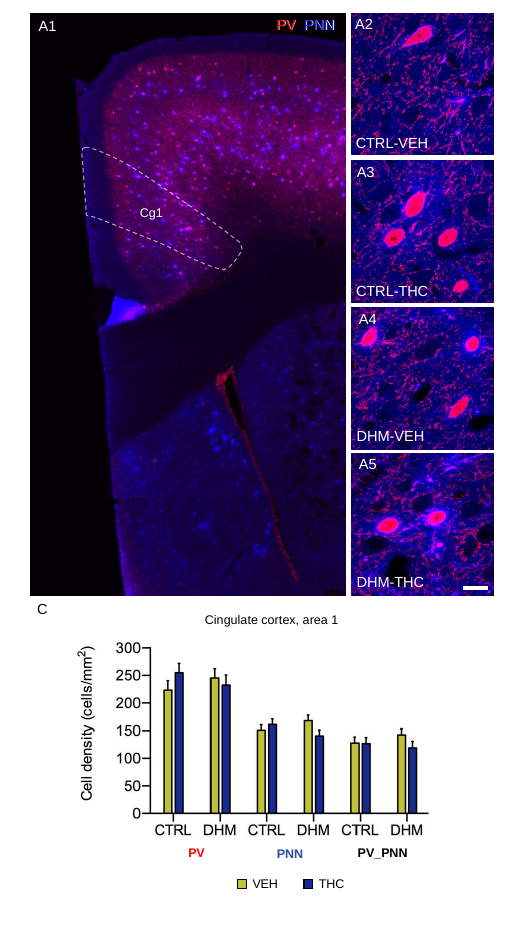

A2
PV PNN
PV PNN
A1
CTRL-VEH
A3
Cg1
CTRL-THC
A4
DHM-VEH
A5
DHM-THC
C
Cingulate cortex, area 1
PV_PNN
PV
PNN
VEH
THC
