# Supplementary material for: Δ-9-Tetrahydrocannabinol treatment during adolescence and alterations in the inhibitory networks of the adult prefrontal cortex in mice subjected to perinatal NMDA receptor antagonist injection and to postweaning social isolation
Source: Transl Psychiatry. 2020 Jun 1;10:177. doi: 10.1038/s41398-020-0853-3 (PMC7266818; doi:10.1038/s41398-020-0853-3)
Supplement: Supplementary file 9 — table S5 [file 41398_2020_853_MOESM9_ESM.pdf]

		SiaII mRNA	SiaIV mRNA	CB1R mRNA	ErbB4 mRNA	GAD67 mRNA	SYN protein	GAD protein	PrL cortex volume	IL cortex volume	Cg1 cortex volume	Sholl sphere_0 micron	Sholl sphere_20 micron	Sholl sphere_40 micron	Sholl sphere_60 micron	Sholl sphere_80 micron	Sholl sphere_100 micron	Sholl sphere_120 micron	Sholl sphere_140 micron	total arborization	dendritic spine density_50 micron	dendritic spine density_100 micron	dendritic spine density_150 micron	total number of dendritic spines	E/I ratio of PrL cortex	density of VGLUT1 expressing puncta in PrL	density of VGAT expressing puncta in PrL	E/I ratio of IL cortex	density of VGLUT1 expressing puncta in IL	density of VGAT expressing puncta in IL	density of CB1R+ puncta on pyramidal neurons of PrL	density of SYN+ puncta on pyramidal neurons of PrL	density of CB1R+ puncta coexpressing SYN on pyramidal neurons of PrL	density of CB1R+ puncta on pyramidal neurons of IL	density of SYN+ puncta on pyramidal neurons of IL	density of CB1R+ puncta coexpressing SYN on pyramidal neurons of IL	density of CB1R+ puncta on pyramidal neurons of Cg1	density of SYN+ puncta on pyramidal neurons of Cg1	density of CB1R+ puncta coexpressing SYN on pyramidal neurons of Cg1	density of PV+ interneurons in PrL	density of PNNs in PrL	density of PV+ interneurons surrounded by PNNs in PrL	density of PV+ interneurons in IL	density of PNNs in IL	density of PV+ interneurons surrounded by PNNs in IL	density of PV+ interneurons in Cg1	density of PNNs in Cg1	density of PV+ interneurons surrounded by PNNs in Cg1
SiaII mRNA	Pearson Correlation	1	.606**	.671**	.596**	-0.029	0.202	-0.021	-0.132	-0.123	-0.417	-.421*	-0.112	0.085	-0.129	-0.156	-0.133	-0.168	0.194	-0.174	-0.218	-0.127	-0.25	-0.224	0.035	0.087	-0.004	-0.154	-0.191	-0.068	-0.14	-0.026	-0.095	0.167	-0.116	0.025	-0.293	.621**	0.403	-0.1	-0.012	0.043	0.008	-0.06	0.214	-0.22	0.174	117,000
	p-value		< 0.001	< 0.001	0.001	0.877	0.488	0.944	0.639	0.663	0.122	0.023	0.555	0.654	0.497	0.41	0.482	0.374	0.305	0.359	0.342	0.585	0.275	0.329	0.882	0.7	0.987	0.506	0.406	0.763	0.545	0.907	0.673	0.458	0.608	0.915	0.197	0.003	0.078	0.674	0.958	0.856	0.974	0.807	0.380	0.338	0.451	0.614
SiaIV mRNA	Pearson Correlation	.606**	1	.835**	.652**	.400*	0.052	-0.036	-0.019	0.002	-0.254	-.470**	-0.159	-0.068	-0.086	-0.108	0.072	-0.045	0.226	-0.145	-0.108	-0.151	-0.194	-0.171	-0.148	0.098	0.148	-0.224	-0.021	0.100	-0.008	-0.05	-0.023	0.023	-0.074	0.053	-0.306	.510*	0.33	-0.109	-0.074	0.01	-0.054	-0.011	0.070	-0.079	-0.097	-0.059
	p-value	< 0.001		< 0.001	< 0.001	0.026	0.854	0.903	0.944	0.993	0.342	0.009	0.394	0.718	0.647	0.564	0.700	0.808	0.222	0.437	0.632	0.503	0.387	0.446	0.511	0.657	0.5	0.316	0.927	0.651	0.972	0.822	0.919	0.916	0.736	0.814	0.167	0.018	0.155	0.638	0.75	0.964	0.817	0.964	0.768	0.727	0.668	0.794
CB1R mRNA	Pearson Correlation	.671**	.835**	1	.842**	0.108	0.089	0.051	0.011	-0.125	-0.32	-.371*	-0.038	0.032	-0.025	-0.046	0.094	-0.028	0.350	-0.024	-0.077	-0.191	-0.12	-0.139	-0.049	0.17	0.157	-0.043	-0.011	0.013	-0.076	0.161	0.127	-0.105	-0.032	0.054	-0.374	0.306	0.092	-0.028	-0.07	-0.144	-0.02	0.026	-0.01	0.09	0.087	0.082
	p-value	< 0.001	< 0.001		< 0.001	0.562	0.753	0.863	0.966	0.644	0.227	0.044	0.839	0.864	0.896	0.805	0.617	0.882	0.054	0.897	0.734	0.393	0.594	0.539	0.827	0.438	0.473	0.851	0.963	0.953	0.736	0.464	0.562	0.635	0.884	0.813	0.087	0.177	0.700	0.906	0.762	0.534	0.933	0.913	0.966	0.691	0.699	0.718
ErbB4 mRNA	Pearson Correlation	.596**	.652**	.842**	1	0.095	0.01	-0.152	-0.025	-0.414	-0.347	-0.184	-0.01	0.08	0.033	-0.099	0.139	0.013	0.330	0.027	-0.038	-0.273	-0.183	-0.189	-0.125	0.006	0.081	-0.048	-0.115	-0.075	0.042	0.173	0.181	-0.265	-0.162	-0.045	-0.086	0.345	0.216	-0.201	-0.054	-0.175	0.174	0.084	0.055	0.231	0.195	0.089
	p-value	0.001	< 0.001	< 0.001		0.609	0.971	0.603	0.926	0.111	0.187	0.331	0.958	0.667	0.86	0.597	0.457	0.946	0.070	0.885	0.865	0.219	0.414	0.399	0.579	0.979	0.715	0.833	0.61	0.735	0.854	0.429	0.407	0.222	0.459	0.842	0.705	0.126	0.359	0.381	0.818	0.449	0.451	0.725	0.817	0.301	0.385	0.694
GAD67 mRNA	Pearson Correlation	-0.029	.400*	0.108	0.095	1	0.201	-0.146	0.088	0.424	0.431	-0.3	-0.28	-.484**	-0.275	-.359*	-0.127	-0.02	0.004	-0.346	0.064	0.017	-0.048	-0.002	-0.112	-0.183	-0.093	0.006	-0.234	-0.258	0.215	-.507*	-0.352	0.011	-0.096	0.132	-0.292	0.183	0.249	0.363	.551**	.589**	0.343	0.424	0.274	0.15	-0.171	-0.079
	p-value	0.877	0.026	0.562	0.609		0.473	0.62	0.746	0.101	0.096	0.101	0.121	0.005	0.128	0.043	0.489	0.915	0.983	0.052	0.770	0.938	0.827	0.992	0.612	0.391	0.664	0.979	0.282	0.224	0.326	0.011	0.092	0.958	0.654	0.549	0.176	0.415	0.276	0.106	0.01	0.005	0.128	0.062	0.242	0.506	0.447	0.727
SYN protein	Pearson Correlation	0.202	0.052	0.089	0.010	0.201	1	0.429	-0.091	0.055	0.348	-0.141	0.197	-0.234	-.581*	-.562*	-.673**	-.600*	-0.214	-.537*	-0.233	-.566*	-0.475	-0.465	-0.036	0.018	0.092	0.063	0.153	0.220	-0.5	-.515*	-.565*	0.125	0.092	0.036	-.579*	0.257	0.070	.521*	0.401	0.479	0.378	0.377	0.324	0.231	0.456	0.356
	p-value	0.488	0.854	0.753	0.971	0.473		0.126	0.747	0.846	0.204	0.631	0.482	0.401	0.023	0.029	0.006	0.018	0.443	0.039	0.403	0.028	0.073	0.081	0.903	0.950	0.744	0.831	0.602	0.431	0.058	0.049	0.028	0.658	0.744	0.899	0.03	0.396	0.828	0.046	0.139	0.071	0.165	0.184	0.259	0.407	0.088	0.192
GAD protein	Pearson Correlation	-0.021	-0.036	0.051	-0.152	-0.146	0.429	1	-0.116	0.29	0.223	0.041	0.281	-0.145	-0.27	0.014	-0.116	-0.234	0.205	-0.039	0.344	-0.014	0.086	0.127	-0.242	0.069	0.401	-0.277	0.242	.573*	-0.295	-0.204	-0.085	0.267	-0.114	0.099	-0.225	0.100	0.110	0.469	0.272	-0.006	0.112	0.314	0.451	0.13	0.183	0.143
	p-value	0.944	0.903	0.863	0.603	0.62	0.126		0.694	0.315	0.444	0.894	0.33	0.622	0.351	0.961	0.693	0.421	0.482	0.896	0.228	0.961	0.77	0.665	0.427	0.815	0.155	0.360	0.425	0.032	0.305	0.485	0.774	0.355	0.698	0.735	0.46	0.757	0.734	0.091	0.346	0.983	0.702	0.296	0.122	0.657	0.531	0.625
PrL cortex volume	Pearson Correlation	-0.132	-0.019	0.011	-0.025	0.088	-0.091	-0.116	1	0.404	.546*	0.265	-.598*	-.565*	-0.317	-0.265	-0.027	-0.123	0.166	-0.241	0.043	0.094	0.066	0.057	-0.046	-0.11	0.048	0.227	0.055	0.047	0.281	0.393	0.483	-0.274	0.013	-0.197	-0.245	-0.271	-0.551	0.098	0.133	0.282	0.198	0.287	0.271	-0.166	-0.3	-0.239
	p-value	0.639	0.944	0.966	0.926	0.746	0.747	0.694		0.121	0.029	0.339	0.014	0.023	0.231	0.321	0.920	0.649	0.539	0.369	0.875	0.729	0.807	0.835	0.870	0.685	0.860	0.416	0.845	0.863	0.291	0.133	0.058	0.304	0.96	0.465	0.38	0.349	0.051	0.718	0.624	0.29	0.463	0.299	0.329	0.539	0.26	0.373
IL cortex volume	Pearson Correlation	-0.123	0.002	-0.125	-0.414	0.424	0.055	0.29	0.404	1	.649**	-0.393	-0.252	-.512*	-0.378	-0.256	-0.427	-0.319	-0.151	-0.368	0.241	0.398	0.451	0.407	0.002	-0.286	-0.232	0.066	-0.08	-0.101	0.013	-0.229	-0.126	0.1	-0.042	-0.035	-0.427	-0.134	-0.357	0.328	0.444	0.369	0.108	0.286	0.323	-0.282	-0.303	-0.231
	p-value	0.663	0.993	0.644	0.111	0.101	0.846	0.315	0.121		0.007	0.148	0.347	0.043	0.148	0.339	0.099	0.228	0.576	0.161	0.369	0.127	0.080	0.118	0.994	0.283	0.388	0.814	0.777	0.71	0.962	0.395	0.642	0.713	0.878	0.897	0.112	0.649	0.231	0.215	0.085	0.16	0.691	0.302	0.241	0.289	0.254	0.388
Cg1 cortex volume	Pearson Correlation	-0.417	-0.254	-0.32	-0.347	0.431	0.348	0.223	.546*	.649**	1	-0.139	-0.441	-.762**	-.603*	-.645**	-.575*	-0.401	-0.184	-.639**	-0.019	-0.025	-0.01	-0.019	0.04	-0.397	-0.209	0.383	-0.068	-0.125	-0.123	-0.297	-0.223	-0.246	-0.103	-0.345	-0.45	-0.073	-0.333	0.479	.509*	.547*	.552*	.535*	.525*	-0.035	-0.103	-0.022
	p-value	0.122	0.342	0.227	0.187	0.096	0.204	0.444	0.029	0.007		0.621	0.087	0.001	0.013	0.007	0.02	0.124	0.494	0.008	0.945	0.926	0.97	0.943	0.888	0.128	0.436	0.158	0.811	0.645	0.65	0.263	0.406	0.359	0.704	0.19	0.092	0.805	0.267	0.06	0.044	0.028	0.027	0.04	0.044	0.897	0.704	0.936
Sholl sphere_0 micron	Pearson Correlation	-.421*	-.470**	-.371*	-0.184	-0.3	-0.141	0.041	0.265	-0.393	-0.139	1	0.004	-0.043	0.003	-0.095	-0.079	-0.22	-0.301	-0.04	-0.166	-0.317	-0.174	-0.24	0.095	-0.139	-0.122	0.118	-0.011	0.04	0.193	.497*	.500*	-0.207	0.032	-0.112	0.218	-0.366	-0.315	-0.227	-0.291	-0.355	-0.196	-0.39	-0.357	0.188	-0.002	-0.078
	p-value	0.023	0.009	0.044	0.331	0.101	0.631	0.894	0.339	0.148	0.621		0.981	0.817	0.985	0.613	0.673	0.233	0.1	0.831	0.461	0.15	0.437	0.283	0.675	0.528	0.58	0.601	0.962	0.857	0.389	0.016	0.015	0.344	0.885	0.62	0.331	0.103	0.176	0.335	0.213	0.125	0.407	0.098	0.133	0.415	0.993	0.738
Sholl sphere_20 micron	Pearson Correlation	-0.112	-0.159	-0.038	-0.01	-0.28	0.197	0.281	-.598*	-0.252	-0.441	0.004	1	.612**	.464**	0.304	0.085	-0.017	0.007	.463**	0.142	-0.162	0.171	0.091	-0.291	-0.05	-0.087	-0.335	-0.072	-0.059	0.158	0.154	0.247	0.086	0.055	0.065	0.257	-0.158	-0.084	-0.139	-0.057	-0.246	-0.314	-0.097	-0.2	0.189	0.164	0.121
	p-value	0.555	0.394	0.839	0.958	0.121	0.482	0.33	0.014	0.347	0.087	0.981		< 0.001	0.008	0.09	0.642	0.927	0.971	0.008	0.518	0.46	0.436	0.679	0.179	0.818	0.685	0.118	0.746	0.786	0.471	0.472	0.245	0.69	0.798	0.77	0.237	0.483	0.716	0.548	0.807	0.283	0.165	0.683	0.397	0.400	0.466	0.590
Sholl sphere_40 micron	Pearson Correlation	0.085	-0.068	0.032	0.08	-.484**	-0.234	-0.145	-.565*	-.512*	-.762**	-0.043	.612**	1	.734**	.654**	.471**	0.33	0.076	.752**	0.023	0.044	0.191	0.127	-0.075	0.119	0.043	-0.292	0.111	0.152	0.105	0.17	0.125	0.235	0.125	0.165	0.411	-0.293	-0.123	-0.385	-0.298	-0.319	-0.358	-0.369	-0.119	-0.188	0.018	0.017
	p-value	0.654	0.718	0.864	0.667	0.005	0.401	0.622	0.023	0.043	0.001	0.817	< 0.001		< 0.001	< 0.001	0.007	0.065	0.679	< 0.001	0.918	0.844	0.384	0.564	0.734	0.581	0.842	0.176	0.614	0.478	0.633	0.426	0.559	0.27	0.56	0.452	0.051	0.186	0.594	0.085	0.189	0.159	0.111	0.109	0.619	0.401	0.937	0.94
Sholl sphere_60 micron	Pearson Correlation	-0.129	-0.086	-0.025	0.033	-0.275	-.581*	-0.27	-0.317	-0.378	-.603*	0.003	.464**	.734**	1	.802**	.771**	.670**	0.346	.921**	0.28	0.336	0.397	0.383	-0.232	0.125	0.218	-0.296	0.101	0.134	0.307	0.121	0.205	0.178	-0.086	0.118	.556**	-0.417	-0.205	-.556**	-0.239	-0.336	-0.425	-0.337	-0.223	-0.281	-0.293	-0.302
	p-value	0.497	0.647	0.896	0.86	0.128	0.023	0.351	0.231	0.148	0.013	0.985	0.008	< 0.001		< 0.001	0,000	< 0.001	0.052	< 0.001	0.196	0.117	0.06	0.071	0.288	0.561	0.306	0.171	0.646	0.532	0.154	0.573	0.337	0.405	0.688	0.592	0.006	0.054	0.374	0.009	0.296	0.136	0.055	0.146	0.344	0.205	0.186	0.171
Sholl sphere_80 micron	Pearson Correlation	-0.156	-0.108	-0.046	-0.099	-.359*	-.562*	0.014	-0.265	-0.256	-.645**	-0.095	0.304	.654**	.802**	1	.854**	.704**	.489**	.904**	0.396	.499*	0.41	.462*	-0.23	.412*	0.379	-0.397	0.338	0.332	0.21	-0.018	0.086	0.371	-0.002	0.271	.512*	-0.366	-0.072	-0.344	-0.313	-0.386	-.449*	-0.299	-0.173	-0.254	-0.337	-0.327
	p-value	0.41	0.564	0.805	0.597	0.043	0.029	0.961	0.321	0.339	0.007	0.613	0.09	< 0.001	< 0.001		0,000	< 0.001	0.005	< 0.001	0.061	0.015	0.052	0.026	0.292	0.046	0.068	0.06	0.114	0.113	0.336	0.932	0.688	0.074	0.994	0.211	0.013	0.093	0.756	0.127	0.166	0.084	0.041	0.201	0.465	0.254	0.125	0.138
Sholl sphere_100 micron	Pearson Correlation	-0.133	0.072	0.094	0.139	-0.127	-.673**	-0.116	-0.027	-0.427	-.575*	-0.079	0.085	.471**	.771**	.854**	1	.820**	.589**	.851**	0.397	.443*	0.327	0.399	-0.211	0.389	.454*	-0.209	0.339	0.307	0.321	-0.02	0.103	0.258	-0.023	0.307	.483*	-0.277	0.046	-.516*	-0.365	-.433*	-0.286	-0.143	-0.029	-0.072	-0.212	-0.206
	p-value	0.482	0.700	0.617	0.457	0.489	0.006	0.693	0.92	0.099	0.02	0.673	0.642	0.007	< 0.001	0,000		< 0.001	< 0.001	< 0.001	0.061	0.034	0.127	0.059	0.335	0.06	0.026	0.339	0.114	0.145	0.136	0.925	0.631	0.224	0.914	0.154	0.019	0.212	0.844	0.017	0.103	0.05	0.208	0.547	0.903	0.749	0.344	0.358
Sholl sphere_120 micron	Pearson Correlation	-0.168	-0.045	-0.028	0.013	-0.02	-.600*	-0.234	-0.123	-0.319	-0.401	-0.22	-0.017	0.33	.670**	.704**	.820**	1	.542**	.737**	0.238	.420*	0.263	0.325	-0.169	.417*	.505*	-0.152	0.281	0.312	0.123	-0.187	-0.131	0.174	0.013	0.311	.465*	-0.22	0.057	-0.388	-0.232	-0.244	-0.117	0.007	-0.006	-0.149	-0.224	-0.19
	p-value	0.374	0.808	0.882	0.946	0.915	0.018	0.421	0.649	0.228	0.124	0.233	0.927	0.065	0,000	< 0.001	< 0.001		0.001	< 0.001	0.273	0.046	0.225	0.13	0.441	0.043	0.012	0.488	0.194	0.138	0.577	0.383	0.542	0.417	0.954	0.148	0.025	0.326	0.805	0.082	0.313	0.285	0.614	0.976	0.978	0.509	0.317	0.397
Sholl sphere_140 micron	Pearson Correlation	0.194	0.226	0.350	0.330	0.004	-0.214	0.205	0.166	-0.151	-0.184	-0.301	0.007	0.076	0.346	.489**	.589**	.542**	1	.472**	0.208	0.183	0.046	0.131	-0.304	0.362	0.393	-0.212	0.125	0.11	0.191	-0.128	0.046	0.237	-0.062	0.228	0.226	0.158	0.248	-0.273	-0.178	-0.272	-0.188	-0.031	0.006	-0.105	-0.169	-0.146
	p-value	0.305	0.222	0.054	0.07	0.983	0.443	0.482	0.539	0.576	0.494	0.100	0.971	0.679	0.052	0.005	< 0.001	0.001		0.006	0.342	0.402	0.835	0.551	0.158	0.082	0.058	0.332	0.569	0.61	0.383	0.551	0.831	0.265	0.774	0.295	0.3	0.483	0.278	0.23	0.439	0.232	0.414	0.897	0.980	0.642	0.453	0.518
total arborization	Pearson Correlation	-0.174	-0.145	-0.024	0.027	-0.346	-.537*	-0.039	-0.241	-0.368	-.639**	-0.04	.463**	.752**	.921**	.904**	.851**	.737**	.472**	1	0.355	0.391	.423*	.429*	-0.246	0.28	0.36	-0.314	0.231	0.285	0.243	0.054	0.15	0.229	-0.059	0.183	.546**	-.459*	-0.189	-.458*	-0.249	-0.387	-0.343	-0.222	-0.12	-0.157	-0.188	-0.195
	p-value	0.359	0.437	0.897	0.885	0.052	0.039	0.896	0.369	0.161	0.008	0.831	0.008	< 0.001	< 0.001	< 0.001	< 0.001	< 0.001	0.006		0.096	0.065	0.044	0.041	0.257	0.184	0.084	0.144	0.288	0.178	0.265	0.801	0.483	0.281	0.783	0.403	0.007	0.032	0.412	0.037	0.277	0.083	0.128	0.348	0.615	0.486	0.402	0.385
dendritic spine density_50 micron	Pearson Correlation	-0.218	-0.108	-0.077	-0.038	0.064	-0.233	0.344	0.043	0.241	-0.019	-0.166	0.142	0.023	0.28	0.396	0.397	0.238	0.208	0.355	1	.775**	.743**	.875**	-0.294	-0.117	0.139	-0.257	0.084	0.103	0.253	-0.011	0.104	0.13	-0.219	-0.102	0.083	-0.294	-0.197	0.186	.454*	0.233	0.121	.501*	0.277	-0.071	-0.075	-0.235
	p-value	0.342	0.632	0.734	0.865	0.770	0.403	0.228	0.875	0.369	0.945	0.461	0.518	0.918	0.196	0.061	0.061	0.273	0.342	0.096		< 0.001	< 0.001	< 0.001	0.184	0.594	0.528	0.249	0.711	0.64	0.245	0.962	0.637	0.556	0.315	0.651	0.713	0.196	0.406	0.42	0.039	0.31	0.6	0.025	0.237	0.753	0.741	0.292
dendritic spine density_100 micron	Pearson Correlation	-0.127	-0.151	-0.191	-0.273	0.017	-.566*	-0.014	0.094	0.398	-0.025	-0.317	-0.162	0.044	0.336	.499*	.443*	.420*	0.183	0.391	.775**	1	.819**	.920**	0.088	0.057	0.105	-0.005	0.132	0.106	0.259	-0.162	-0.05	0.274	-0.297	-0.117	0.098	-0.332	-0.196	0.129	0.343	0.224	0.104	0.314	0.258	-0.251	-0.191	-0.223
	p-value	0.585	0.503	0.393	0.219	0.938	0.028	0.961	0.729	0.127	0.926	0.150	0.460	0.844	0.117	0.015	0.034	0.046	0.402	0.065	< 0.001		< 0.001	< 0.001	0.696	0.796	0.634	0.981	0.559	0.63	0.233	0.46	0.822	0.206	0.168	0.605	0.663	0.141	0.407	0.577	0.128	0.330	0.655	0.178	0.272	0.259	0.394	0.318
dendritic spine density_150 micron	Pearson Correlation	-0.25	-0.194	-0.12	-0.183	-0.048	-0.475	0.086	0.066	0.451	-0.01	-0.174	0.171	0.191	0.397	0.41	0.327	0.263	0.046	.423*	.743**	.819**	1	.959**	0.043	-0.216	-0.133	0.007	0,000	-0.056	0.302	0.134	0.29	0.291	-0.144	0.03	0.221	-.456*	-0.295	0.04	0.31	0.085	-0.081	0.168	0.039	-0.056	-0.034	-0.055
	p-value	0.275	0.387	0.594	0.414	0.827	0.073	0.770	0.807	0.080	0.97	0.437	0.436	0.384	0.060	0.052	0.127	0.225	0.835	0.044	< 0.001	< 0.001		< 0.001	0.848	0.322	0.546	0.974	0.998	0.801	0.161	0.543	0.18	0.178	0.511	0.896	0.323	0.038	0.206	0.864	0.172	0.713	0.728	0.479	0.869	0.806	0.881	0.806
total number of dendritic spines	Pearson Correlation	-0.224	-0.171	-0.139	-0.189	-0.002	-0.465	0.127	0.057	0.407	-0.019	-0.24	0.091	0.127	0.383	.462*	0.399	0.325	0.131	.429*	.875**	.920**	.959**	1	-0.029	-0.137	-0.011	-0.069	0.052	0.024	0.293	0.021	0.162	0.269	-0.206	-0.042	0.169	-0.399	-0.252	0.111	0.38	0.170	0.014	0.308	0.159	-0.124	-0.091	-0.149
	p-value	0.329	0.446	0.539	0.399	0.992	0.081	0.665	0.835	0.118	0.943	0.283	0.679	0.564	0.071	0.026	0.059	0.13	0.551	0.041	< 0.001	< 0.001	< 0.001		0.9	0.534	0.96	0.759	0.819	0.912	0.175	0.923	0.459	0.214	0.345	0.853	0.451	0.073	0.283	0.633	0.09	0.461	0.952	0.187	0.504	0.584	0.687	0.508
E/I ratio of PrL cortex	Pearson Correlation	0.035	-0.148	-0.049	-0.125	-0.112	-0.036	-0.242	-0.046	0.002	0.04	0.095	-0.291	-0.075	-0.232	-0.23	-0.211	-0.169	-0.304	-0.246	-0.294	0.088	0.043	-0.029	1	0.029	-.437*	.855**	0.081	-0.239	0.191	-0.233	-0.149	0.301	0.089	0.085	-0.332	-0.018	-0.08	0.086	-0.115	-0.074	0.165	-0.217	-0.164	0.342	0.375	.474*
	p-value	0.882	0.511	0.827	0.579	0.612	0.903	0.427	0.87	0.994	0.888	0.675	0.179	0.734	0.288	0.292	0.335	0.441	0.158	0.257	0.184	0.696	0.848	0.9		0.897	0.037	< 0.001	0.72	0.272	0.395	0.285	0.499	0.163	0.685	0.706	0.131	0.94	0.738	0.718	0.63	0.757	0.488	0.372	0.501	0.129	0.094	0.03
density of VGLUT1 expressing puncta in PrL	Pearson Correlation	0.087	0.098	0.170	0.006	-0.183	0.018	0.069	-0.11	-0.286	-0.397	-0.139	-0.05	0.119	0.125	.412*	0.389	.417*	0.362	0.28	-0.117	0.057	-0.216	-0.137	0.029	1	.751**	0.057	.796**	.616**	-0.126	-0.248	-0.288	0.284	0.215	.441*	0.03	-0.103	0.216	-0.109	-0.248	-0.163	-0.162	0.009	0.042	-0.08	0.099	0.058
	p-value	0.700	0.657	0.438	0.979	0.391	0.95	0.815	0.685	0.283	0.128	0.528	0.818	0.581	0.561	0.046	0.06	0.043	0.082	0.184	0.594	0.796	0.322	0.534	0.897		0,000	0.798	0,000	0.001	0.565	0.242	0.173	0.179	0.312	0.035	0.89	0.65	0.348	0.638	0.279	0.480	0.483	0.97	0.861	0.724	0.662	0.797
density of VGAT expressing puncta in PrL	Pearson Correlation	-0.004	0.148	0.157	0.081	-0.093	0.092	0.401	0.048	-0.232	-0.209	-0.122	-0.087	0.043	0.218	0.379	.454*	.505*	0.393	0.36	0.139	0.105	-0.133	-0.011	-.437*	.751**	1	-0.329	.656**	.825**	-0.278	-0.137	-0.251	-0.013	-0.064	0.081	0.116	-0.176	0.028	-0.11	-0.008	0.018	-0.052	0.243	0.182	-0.234	-0.04	-0.141
	p-value	0.987	0.500	0.473	0.715	0.664	0.744	0.155	0.86	0.388	0.436	0.58	0.685	0.842	0.306	0.068	0.026	0.012	0.058	0.084	0.528	0.634	0.546	0.96	0.037	< 0.001		0.125	0.001	0,000	0.199	0.524	0.237	0.952	0.767	0.712	0.6	0.433	0.903	0.636	0.973	0.939	0.823	0.303	0.443	0.294	0.858	0.530
E/I ratio of IL cortex	Pearson Correlation	-0.154	-0.224	-0.043	-0.048	0.006	0.063	-0.277	0.227	0.066	0.383	0.118	-0.335	-0.292	-0.296	-0.397	-0.209	-0.152	-0.212	-0.314	-0.257	-0.005	0.007	-0.069	.855**	0.057	-0.329	1	0.212	-0.293	0.254	-0.164	-0.043	0.115	0.108	0.133	-0.394	-0.107	-0.123	0.101	0.053	0.044	0.273	0.040	-0.023	0.41	0.384	.451*
	p-value	0.506	0.316	0.851	0.833	0.979	0.831	0.36	0.416	0.814	0.158	0.601	0.118	0.176	0.171	0.06	0.339	0.488	0.332	0.144	0.249	0.981	0.974	0.759	< 0.001	0.798	0.125		0.342	0.175	0.254	0.454	0.845	0.6	0.624	0.556	0.07	0.646	0.605	0.673	0.823	0.853	0.244	0.871	0.927	0.065	0.085	0.04
density of VGLUT1 expressing puncta in IL	Pearson Correlation	-0.191	-0.021	-0.011	-0.115	-0.234	0.153	0.242	0.055	-0.08	-0.068	-0.011	-0.072	0.111	0.101	0.338	0.339	0.281	0.125	0.231	0.084	0.132	0,000	0.052	0.081	.796**	.656**	0.212	1	.734**	-0.116	-0.19	-0.202	0.34	0.372	.466*	-0.003	-0.231	0.086	-0.151	-0.131	-0.045	-0.086	0.081	0.072	-0.158	0.12	0.004
	p-value	0.406	0.927	0.963	0.610	0.282	0.602	0.425	0.845	0.777	0.811	0.962	0.746	0.614	0.646	0.114	0.114	0.194	0.569	0.288	0.711	0.559	0.998	0.819	0.72	< 0.001	0.001	0.342		< 0.001	0.607	0.386	0.354	0.113	0.081	0.029	0.989	0.314	0.72	0.526	0.582	0.85	0.719	0.74	0.764	0.495	0.604	0.986
density of VGAT expressing puncta in IL	Pearson Correlation	-0.068	0.100	0.013	-0.075	-0.258	0.22	.573*	0.047	-0.101	-0.125	0.04	-0.059	0.152	0.134	0.332	0.307	0.312	0.11	0.285	0.103	0.106	-0.056	0.024	-0.239	.616**	.825**	-0.293	.734**	1	-0.308	-0.132	-0.229	0.132	0.025	0.029	0.092	-0.187	-0.035	-0.082	-0.06	0.039	-0.013	0.100	0.165	-0.248	0.014	-0.077
	p-value	0.763	0.651	0.953	0.735	0.224	0.431	0.032	0.863	0.71	0.645	0.857	0.786	0.478	0.532	0.113	0.145	0.138	0.61	0.178	0.64	0.63	0.801	0.912	0.272	0.001	0,000	0.175	< 0.001		0.153	0.537	0.283	0.539	0.907	0.897	0.675	0.404	0.882	0.722	0.797	0.867	0.957	0.676	0.487	0.265	0.95	0.732
density of CB1R+ puncta on pyramidal neurons of PrL	Pearson Correlation	-0.14	-0.008	-0.076	0.042	0.215	-0.5	-0.295	0.281	0.013	-0.123	0.193	0.158	0.105	0.307	0.21	0.321	0.123	0.191	0.243	0.253	0.259	0.302	0.293	0.191	-0.126	-0.278	0.254	-0.116	-0.308	1	0.047	.502*	0.333	-0.237	0.208	0.12	-0.2	-0.094	-0.386	0.043	-0.012	-0.338	-0.106	-0.153	0.191	-0.129	-0.126
	p-value	0.545	0.972	0.736	0.854	0.326	0.058	0.305	0.291	0.962	0.65	0.389	0.471	0.633	0.154	0.336	0.136	0.577	0.383	0.265	0.245	0.233	0.161	0.175	0.395	0.565	0.199	0.254	0.607	0.153		0.83	0.015	0.12	0.277	0.353	0.594	0.384	0.693	0.084	0.853	0.959	0.134	0.656	0.52	0.394	0.568	0.576
density of SYN+ puncta on pyramidal neurons of PrL	Pearson Correlation	-0.026	-0.05	0.161	0.173	-.507*	-.515*	-0.204	0.393	-0.229	-0.297	.497*	0.154	0.17	0.121	-0.018	-0.02	-0.187	-0.128	0.054	-0.011	-0.162	0.134	0.021	-0.233	-0.248	-0.137	-0.164	-0.19	-0.132	0.047	1	.837**	-0.388	0.165	-0.16	0.327	-0.191	-0.309	-0.323	-0.432	-0.432	-.476*	-0.347	-0.435	-0.124	-0.19	-0.177
	p-value	0.907	0.822	0.464	0.429	0.011	0.049	0.485	0.133	0.395	0.263	0.016	0.472	0.426	0.573	0.932	0.925	0.383	0.551	0.801	0.962	0.46	0.543	0.923	0.285	0.242	0.524	0.454	0.386	0.537	0.83		< 0.001	0.061	0.441	0.467	0.128	0.395	0.173	0.153	0.051	0.05	0.029	0.134	0.055	0.581	0.398	0.429
density of CB1R+ puncta coexpressing SYN on pyramidal neurons of PrL	Pearson Correlation	-0.095	-0.023	0.127	0.181	-0.352	-.565*	-0.085	0.483	-0.126	-0.223	.500*	0.247	0.125	0.205	0.086	0.103	-0.131	0.046	0.15	0.104	-0.05	0.29	0.162	-0.149	-0.288	-0.251	-0.043	-0.202	-0.229	.502*	.837**	1	-0.109	-0.043	-0.06	0.333	-0.186	-0.223	-0.422	-0.276	-0.367	-.526*	-0.289	-0.379	0.06	-0.183	-0.171
	p-value	0.673	0.919	0.562	0.407	0.092	0.028	0.774	0.058	0.642	0.406	0.015	0.245	0.559	0.337	0.688	0.631	0.542	0.831	0.483	0.637	0.822	0.18	0.459	0.499	0.173	0.237	0.845	0.354	0.283	0.015	< 0.001		0.612	0.841	0.786	0.12	0.407	0.331	0.057	0.226	0.101	0.014	0.216	0.099	0.791	0.416	0.448
density of CB1R+ puncta on pyramidal neurons of IL	Pearson Correlation	0.167	0.023	-0.105	-0.265	0.011	0.125	0.267	-0.274	0.1	-0.246	-0.207	0.086	0.235	0.178	0.371	0.258	0.174	0.237	0.229	0.13	0.274	0.291	0.269	0.301	0.284	-0.013	0.115	0.34	0.132	0.333	-0.388	-0.109	1	0.239	.649**	0.074	0.111	0.405	-0.209	-0.011	-0.009	-0.358	-0.281	-0.031	-0.062	0.249	0.23
	p-value	0.458	0.916	0.635	0.222	0.958	0.658	0.355	0.304	0.713	0.359	0.344	0.69	0.27	0.405	0.074	0.224	0.417	0.265	0.281	0.556	0.206	0.178	0.214	0.163	0.179	0.952	0.6	0.113	0.539	0.12	0.061	0.612		0.26	0.001	0.736	0.624	0.068	0.363	0.964	0.968	0.112	0.231	0.896	0.783	0.263	0.303
density of SYN+ puncta on pyramidal neurons of IL	Pearson Correlation	-0.116	-0.074	-0.032	-0.162	-0.096	0.092	-0.114	0.013	-0.042	-0.103	0.032	0.055	0.125	-0.086	-0.002	-0.023	0.013	-0.062	-0.059	-0.219	-0.297	-0.144	-0.206	0.089	0.215	-0.064	0.108	0.372	0.025	-0.237	0.165	-0.043	0.239	1	.728**	0.05	0.141	0.328	-0.099	-.560**	-0.34	-0.354	-0.43	-0.422	-0.115	0.085	0.134
	p-value	0.608	0.736	0.884	0.459	0.654	0.744	0.698	0.96	0.878	0.704	0.885	0.798	0.56	0.688	0.994	0.914	0.954	0.774	0.783	0.315	0.168	0.511	0.345	0.685	0.312	0.767	0.624	0.081	0.907	0.277	0.441	0.841	0.26		0.001	0.819	0.532	0.147	0.669	0.008	0.132	0.115	0.059	0.064	0.61	0.707	0.553
density of CB1R+ puncta coexpressing SYN on pyramidal neurons of IL	Pearson Correlation	0.025	0.053	0.054	-0.045	0.132	0.036	0.099	-0.197	-0.035	-0.345	-0.112	0.065	0.165	0.118	0.271	0.307	0.311	0.228	0.183	-0.102	-0.117	0.03	-0.042	0.085	.441*	0.081	0.133	.466*	0.029	0.208	-0.16	-0.06	.649**	.728**	1	0.134	0.209	.637**	-0.37	-0.441	-0.43	-.561**	-0.384	-0.278	0.027	0.195	0.184
	p-value	0.915	0.814	0.813	0.842	0.549	0.899	0.735	0.465	0.897	0.19	0.62	0.77	0.452	0.592	0.211	0.154	0.148	0.295	0.403	0.651	0.605	0.896	0.853	0.706	0.035	0.712	0.556	0.029	0.897	0.353	0.467	0.786	0.001	< 0.001		0.552	0.364	0.003	0.109	0.052	0.058	0.01	0.105	0.25	0.908	0.398	0.424
density of CB1R+ puncta on pyramidal neurons of Cg1	Pearson Correlation	-0.293	-0.306	-0.374	-0.086	-0.292	-.579*	-0.225	-0.245	-0.427	-0.45	0.218	0.257	0.411	.556**	.512*	.483*	.465*	0.226	.546**	0.083	0.098	0.221	0.169	-0.332	0.03	0.116	-0.394	-0.003	0.092	0.12	0.327	0.333	0.074	0.05	0.134	1	-0.105	0.392	-.584**	-.529*	-.550*	-0.388	-0.394	-0.28	0.007	-0.045	-0.044
	p-value	0.197	0.167	0.087	0.705	0.176	0.03	0.46	0.38	0.112	0.092	0.331	0.237	0.051	0.006	0.013	0.019	0.025	0.3	0.007	0.713	0.663	0.323	0.451	0.131	0.89	0.6	0.07	0.989	0.675	0.594	0.128	0.12	0.736	0.819	0.552		0.642	0.079	0.007	0.017	0.012	0.091	0.086	0.246	0.976	0.848	0.85
density of SYN+ puncta on pyramidal neurons of Cg1	Pearson Correlation	.621**	.510*	0.306	0.345	0.183	0.257	0.1	-0.271	-0.134	-0.073	-0.366	-0.158	-0.293	-0.417	-0.366	-0.277	-0.22	0.158	-.459*	-0.294	-0.332	-.456*	-0.399	-0.018	-0.103	-0.176	-0.107	-0.231	-0.187	-0.2	-0.191	-0.186	0.111	0.141	0.209	-0.105	1	.778**	-0.093	-0.23	-0.12	0.097	-0.066	0.092	0.026	0.187	0.223
	p-value	0.003	0.018	0.177	0.126	0.415	0.396	0.757	0.349	0.649	0.805	0.103	0.483	0.186	0.054	0.093	0.212	0.326	0.483	0.032	0.196	0.141	0.038	0.073	0.94	0.65	0.433	0.646	0.314	0.404	0.384	0.395	0.407	0.624	0.532	0.364	0.642		< 0.001	0.703	0.344	0.624	0.692	0.787	0.716	0.913	0.430	0.345
density of CB1R+ puncta coexpressing SYN on pyramidal neurons of Cg1	Pearson Correlation	0.403	0.330	0.092	0.216	0.249	0.07	0.11	-0.551	-0.357	-0.333	-0.315	-0.084	-0.123	-0.205	-0.072	0.046	0.057	0.248	-0.189	-0.197	-0.196	-0.295	-0.252	-0.08	0.216	0.028	-0.123	0.086	-0.035	-0.094	-0.309	-0.223	0.405	0.328	.637**	0.392	.778**	1	-0.266	-0.363	-0.279	-0.016	-0.128	0.123	0.111	0.304	0.333
	p-value	0.078	0.155	0.700	0.359	0.276	0.828	0.734	0.051	0.231	0.267	0.176	0.716	0.594	0.374	0.756	0.844	0.805	0.278	0.412	0.406	0.407	0.206	0.283	0.738	0.348	0.903	0.605	0.72	0.882	0.693	0.173	0.331	0.068	0.147	0.003	0.079	< 0.001		0.286	0.139	0.261	0.949	0.613	0.637	0.65	0.205	0.163
density of PV+ interneurons in PrL	Pearson Correlation	-0.1	-0.109	-0.028	-0.201	0.363	.521*	0.469	0.098	0.328	0.479	-0.227	-0.139	-0.385	-.556**	-0.344	-.516*	-0.388	-0.273	-.458*	0.186	0.129	0.04	0.111	0.086	-0.109	-0.11	0.101	-0.151	-0.082	-0.386	-0.323	-0.422	-0.209	-0.099	-0.37	-.584**	-0.093	-0.266	1	.560**	.585**	.585**	.580**	0.413	0.132	-0.002	0.095
	p-value	0.674	0.638	0.906	0.381	0.106	0.046	0.091	0.718	0.215	0.06	0.335	0.548	0.085	0.009	0.127	0.017	0.082	0.230	0.037	0.420	0.577	0.864	0.633	0.718	0.638	0.636	0.673	0.526	0.722	0.084	0.153	0.057	0.363	0.669	0.109	0.007	0.703	0.286		0.008	0.005	0.005	0.007	0.07	0.568	0.992	0.683
density of PNNs in PrL	Pearson Correlation	-0.012	-0.074	-0.07	-0.054	.551**	0.401	0.272	0.133	0.444	.509*	-0.291	-0.057	-0.298	-0.239	-0.313	-0.365	-0.232	-0.178	-0.249	.454*	0.343	0.31	0.380	-0.115	-0.248	-0.008	0.053	-0.131	-0.06	0.043	-0.432	-0.276	-0.011	-.560**	-0.441	-.529*	-0.23	-0.363	.560**	1	.839**	.529*	.753**	.585**	-0.074	0.016	-0.08
	p-value	0.958	0.750	0.762	0.818	0.010	0.139	0.346	0.624	0.085	0.044	0.213	0.807	0.189	0.296	0.166	0.103	0.313	0.439	0.277	0.039	0.128	0.172	0.090	0.630	0.279	0.973	0.823	0.582	0.797	0.853	0.051	0.226	0.964	0.008	0.052	0.017	0.344	0.139	0.008		< 0.001	0.014	< 0.001	0.007	0.748	0.944	0.729
density of PV+ interneurons surrounded by PNNs in PrL	Pearson Correlation	0.043	0.010	-0.144	-0.175	.589**	0.479	-0.006	0.282	0.369	.547*	-0.355	-0.246	-0.319	-0.336	-0.386	-.433*	-0.244	-0.272	-0.387	0.233	0.224	0.085	0.170	-0.074	-0.163	0.018	0.044	-0.045	0.039	-0.012	-0.432	-0.367	-0.009	-0.34	-0.43	-.550*	-0.12	-0.279	.585**	.839**	1	.487*	.658**	.510*	-0.236	-0.119	-0.157
	p-value	0.856	0.964	0.534	0.449	0.005	0.071	0.983	0.290	0.160	0.028	0.125	0.283	0.159	0.136	0.084	0.05	0.285	0.232	0.083	0.310	0.330	0.713	0.461	0.757	0.48	0.939	0.853	0.85	0.867	0.959	0.05	0.101	0.968	0.132	0.058	0.012	0.624	0.261	0.005	< 0.001		0.025	0.002	0.022	0.302	0.607	0.498
density of PV+ interneurons in IL	Pearson Correlation	0.008	-0.054	-0.02	0.174	0.343	0.378	0.112	0.198	0.108	.552*	-0.196	-0.314	-0.358	-0.425	-.449*	-0.286	-0.117	-0.188	-0.343	0.121	0.104	-0.081	0.014	0.165	-0.162	-0.052	0.273	-0.086	-0.013	-0.338	-.476*	-.526*	-0.358	-0.354	-.561**	-0.388	0.097	-0.016	.585**	.529*	.487*	1	.745**	.769**	0.272	0.297	0.275
	p-value	0.974	0.817	0.933	0.451	0.128	0.165	0.702	0.463	0.691	0.027	0.407	0.165	0.111	0.055	0.041	0.208	0.614	0.414	0.128	0.600	0.655	0.728	0.952	0.488	0.483	0.823	0.244	0.719	0.957	0.134	0.029	0.014	0.112	0.115	0.01	0.091	0.692	0.949	0.005	0.014	0.025		< 0.001	0,000	0.233	0.192	0.228
density of PNNs in IL	Pearson Correlation	-0.06	-0.011	0.026	0.084	0.424	0.377	0.314	0.287	0.286	.535*	-0.39	-0.097	-0.369	-0.337	-0.299	-0.143	0.007	-0.031	-0.222	.501*	0.314	0.168	0.308	-0.217	0.009	0.243	0.04	0.081	0.1	-0.106	-0.347	-0.289	-0.281	-0.43	-0.384	-0.394	-0.066	-0.128	.580**	.753**	.658**	.745**	1	.802**	0.151	0.179	0.112
	p-value	0.807	0.964	0.913	0.725	0.062	0.184	0.296	0.299	0.302	0.04	0.098	0.683	0.109	0.146	0.201	0.547	0.976	0.897	0.348	0.025	0.178	0.479	0.187	0.372	0.97	0.303	0.871	0.74	0.676	0.656	0.134	0.216	0.231	0.059	0.105	0.086	0.787	0.613	0.007	< 0.001	0.002	< 0.001		< 0.001	0.526	0.451	0.638
density of PV+ interneurons surrounded by PNNs in IL	Pearson Correlation	0.214	0.070	-0.01	0.055	0.274	0.324	0.451	0.271	0.323	.525*	-0.357	-0.2	-0.119	-0.223	-0.173	-0.029	-0.006	0.006	-0.12	0.277	0.258	0.039	0.159	-0.164	0.042	0.182	-0.023	0.072	0.165	-0.153	-0.435	-0.379	-0.031	-0.422	-0.278	-0.28	0.092	0.123	0.413	.585**	.510*	.769**	.802**	1	0.002	0.215	0.201
	p-value	0.380	0.768	0.966	0.817	0.242	0.259	0.122	0.329	0.241	0.044	0.133	0.397	0.619	0.344	0.465	0.903	0.978	0.98	0.615	0.237	0.272	0.869	0.504	0.501	0.861	0.443	0.927	0.764	0.487	0.52	0.055	0.099	0.896	0.064	0.25	0.246	0.716	0.637	0.070	0.007	0.022	< 0.001	< 0.001		0.992	0.363	0.395
density of PV+ interneurons in Cg1	Pearson Correlation	-0.22	-0.079	0.09	0.231	0.150	0.231	0.13	-0.166	-0.282	-0.035	0.188	0.189	-0.188	-0.281	-0.254	-0.072	-0.149	-0.105	-0.157	-0.071	-0.251	-0.056	-0.124	0.342	-0.08	-0.234	0.41	-0.158	-0.248	0.191	-0.124	0.06	-0.062	-0.115	0.027	0.007	0.026	0.111	0.132	-0.074	-0.236	0.272	0.151	0.002	1	.651**	.699**
	p-value	0.338	0.727	0.691	0.301	0.506	0.407	0.657	0.539	0.289	0.897	0.415	0.400	0.401	0.205	0.254	0.749	0.509	0.642	0.486	0.753	0.259	0.806	0.584	0.129	0.724	0.294	0.065	0.495	0.265	0.394	0.581	0.791	0.783	0.610	0.908	0.976	0.913	0.65	0.568	0.748	0.302	0.233	0.526	0.992		0.001	< 0.001
density of PNNs in Cg1	Pearson Correlation	0.174	-0.097	0.087	0.195	-0.171	0.456	0.183	-0.3	-0.303	-0.103	-0.002	0.164	0.018	-0.293	-0.337	-0.212	-0.224	-0.169	-0.188	-0.075	-0.191	-0.034	-0.091	0.375	0.099	-0.04	0.384	0.12	0.014	-0.129	-0.19	-0.183	0.249	0.085	0.195	-0.045	0.187	0.304	-0.002	0.016	-0.119	0.297	0.179	0.215	.651**	1	.920**
	p-value	0.451	0.668	0.699	0.385	0.447	0.088	0.531	0.26	0.254	0.704	0.993	0.466	0.937	0.186	0.125	0.344	0.317	0.453	0.402	0.741	0.394	0.881	0.687	0.094	0.662	0.858	0.085	0.604	0.950	0.568	0.398	0.416	0.263	0.707	0.398	0.848	0.430	0.205	0.992	0.944	0.607	0.192	0.451	0.363	0.001		< 0.001
density of PV+ interneurons surrounded by PNNs in Cg1	Pearson Correlation	0.117	-0.059	0.082	0.089	-0.079	0.356	0.143	-0.239	-0.231	-0.022	-0.078	0.121	0.017	-0.302	-0.327	-0.206	-0.19	-0.146	-0.195	-0.235	-0.223	-0.055	-0.149	.474*	0.058	-0.141	.451*	0.004	-0.077	-0.126	-0.177	-0.171	0.230	0.134	0.184	-0.044	0.223	0.333	0.095	-0.08	-0.157	0.275	0.112	0.201	.699**	.920**	1
	p-value	0.614	0.794	0.718	0.694	0.727	0.192	0.625	0.373	0.388	0.936	0.738	0.590	0.94	0.171	0.138	0.358	0.397	0.518	0.385	0.292	0.318	0.806	0.508	0.03	0.797	0.53	0.04	0.986	0.732	0.576	0.429	0.448	0.303	0.553	0.424	0.850	0.345	0.163	0.683	0.729	0.498	0.228	0.638	0.395	< 0.001	< 0.001	
